# Impact of a homestead food production program on women's empowerment: Pro-WEAI results from the FAARM trial in Bangladesh

**DOI:** 10.1016/j.worlddev.2022.106001

**Published:** 2022-10

**Authors:** Jillian L. Waid, Amanda S. Wendt, Sheela S. Sinharoy, Abdul Kader, Sabine Gabrysch

**Affiliations:** a Research Department 2, Potsdam Institute for Climate Impact Research (PIK), Member of the Leibniz Association, Potsdam, Germany; b Heidelberg Institute of Global Health, Heidelberg University, Heidelberg, Germany; c Bangladesh Country Office, Helen Keller International, Dhaka, Bangladesh; d Hubert Department of Global Health and Gangarosa Department of Environmental Health, Rollins School of Public Health, Emory University, Atlanta, GA, USA; e Institute of Public Health, Charité – Universitätsmedizin Berlin, corporate member of Freie Universität Berlin and Humboldt-Universität zu Berlin, Berlin, Germany

**Keywords:** Agriculture, Agency, Gender equity, Homestead food production, Self-efficacy, Women’s groups, WEAI, Women's Empowerment in Agriculture Index, FAARM, Food and Agricultural Approaches to Reducing Malnutrition, pro-WEAI, project-level Women's Empowerment in Agriculture Index, SDG, Sustainable Development Goal, OR, Odds Ratio, GAAP2, Gender, Agriculture, and Assets Project–Phase 2, IFPRI, International Food Policy Research Institute, RCT, Randomized controlled trial, DHS, Demographic and Health Survey, ODK, Open Data Kit, 3DE, Three domains of empowerment, GPI, Gender Parity Index

## Abstract

Nutrition-sensitive agricultural programs have the potential to improve women's and children's nutrition, along with women's empowerment. The project-level Women's Empowerment in Agriculture Index (pro-WEAI) aims to standardize the measurement of women's agency and enable the assessment of impact over typical project timelines. Within the Food and Agricultural Approaches to Reducing Malnutrition (FAARM) cluster-randomized controlled trial in rural Habiganj, Sylhet, Bangladesh, we examined quantitative pro-WEAI data collected from a subsample of trial participants and their husbands (n = 885) approximately four months after the end of the intervention. We evaluated the impact of a three-year homestead food production program on men’s and women's agency separately by pro-WEAI domain and indicator, using multilevel logistic and linear regression. We show that women in the FAARM intervention group had levels of agency similar to men and much higher than women in the control group (Odds Ratio [OR] 7.7, p < 0.001), corresponding to better gender equity in intervention areas (OR 3.5, p < 0.001). The higher levels of agency among intervention women were driven by greater intrinsic and collective agency but not by instrumental agency. Compared to controls, more women in the intervention group found intimate partner violence unacceptable (OR 3.5, p < 0.001), had greater ownership of assets (OR 2.6, p = 0.001), better control of income (OR 1.8, p = 0.042), higher levels of group membership (OR 14.0, p < 0.001), and membership in groups they considered influential (OR 166.8, p < 0.001). Self-efficacy was greater in intervention areas for both women (OR 3.2, p < 0.001) and men (OR 2.3, p = 0.002). Our results contribute to the development of benchmarks for interpreting pro-WEAI scores across programs. Our assessment of the impact of a homestead food production program on women's agency provides additional rationale for women-led agricultural projects. We plan to build on these findings by examining the role of improved women's agency on the pathway from the intervention to nutritional impacts.

## Highlights

• We evaluated women's and men's agency in agriculture in an RCT in rural Bangladesh.
• The intervention supported gardening and poultry rearing through farmer groups.
• We used the project-level Women's Empowerment in Agriculture Index, a new method.
• Intervention women had greater intrinsic and collective, but not instrumental, agency.
• The intervention resulted in higher self-efficacy among both women and men.

## 1 Introduction

Improving gender equality is of intrinsic importance throughout the world, as reflected in Sustainable Development Goal (SDG) 5 “Achieve gender equality and empower all women and girls” (United Nations Sustainable Development Goals, 2015). Women's empowerment is also considered key to achieving several other SDGs, including SDG2 “End hunger, achieve food security and improved nutrition and promote sustainable agriculture.” One pathway through which women's empowerment may influence SDG2 is women's influence on intra-household food or resource allocation, leading to larger investments in the family's health and nutrition (Kadiyala et al., 2014, Ruel and Alderman, 2013).

Empirical evidence for these links is primarily based on cross-sectional studies that have found positive associations between dimensions of empowerment and women's diets (Malapit and Quisumbing, 2015, Amugsi et al., 2016, Sinharoy et al., 2018, Sinharoy et al., 2019, Jones et al., 2020, Kassie et al., 2020), infant and child feeding practices (Bose, 2011, Malapit and Quisumbing, 2015, Malapit et al., 2015), and child nutritional status (De Silva and Harpham, 2007, Shroff et al., 2009, Shroff et al., 2011, Cunningham et al., 2015, Aguayo et al., 2016). However, the relationship between women's empowerment and child nutrition has been called into question by a recent systematic review, which found mixed results (Santoso et al., 2019). All studies included in the review were cross-sectional and the authors recommended further research using longitudinal studies and randomized trials. Comparing empowerment indicators across studies also proved challenging. The authors cataloged over 200 empowerment indicators but noted that even when the same dimension of empowerment was assessed, differences in measurement tools inhibited comparisons between studies. Theory-driven measurement and comparable indicators across contexts will be essential to further understanding the critical links between women's empowerment and women's and children's nutrition and health.

The Women's Empowerment in Agriculture Index (WEAI) was developed in 2012 to measure empowerment among agricultural households across USAID's Feed the Future projects (Alkire et al., 2013). The index aims to measure women's empowerment using Kabeer's definition of empowerment as “the expansion in people's ability to make strategic life choices in a context where this ability was previously denied to them” (Kabeer, 2001). Kabeer conceptualizes empowerment as consisting of three domains: resources, agency, and achievements. The WEAI focused primarily on agency – which Kabeer defines as “the ability to define one's goals and act upon them” – in the agricultural context.

Over time, demand increased for a more sensitive indicator of women's agency to support program monitoring and evaluation efforts (Malapit et al., 2019) and to differentiate between strategies that empower women in contrast to only reaching or benefitting them (Johnson et al., 2018). In response, the International Food Policy Research Institute (IFPRI) developed the project-level WEAI (pro-WEAI) through the Gender, Agriculture, and Assets Project – Phase 2 (GAAP2). The pro-WEAI contains 12 indicators covering three domains of agency: intrinsic (power within), instrumental (power to), and collective agency (power with). These themes of power (Rowlands, 1997, Kabeer, 1994) were present in past WEAI versions but made more explicit in the pro-WEAI (Ibrahim and Alkire, 2007, Malapit et al., 2019). Thirteen projects across Africa and South Asia, as part of the GAAP2 portfolio, provided feedback on the development of components and piloted the pro-WEAI modules. Projects contributed to discussions on pro-WEAI module development and field-tested new qualitative and quantitative tools (International Food Policy Research Institute (IFPRI), 2020).

Though many nutrition-sensitive agriculture programs assume that they will affect empowerment outcomes, few have measured agency in a randomized controlled trial (RCT). The Food and Agricultural Approaches to Reducing Malnutrition (FAARM) trial is one of the first RCTs to evaluate program impacts on men's and women's agency using the pro-WEAI tool. In this manuscript, we start by describing the methods used and explaining the possible means by which the FAARM trial could have impacted women's agency. We then examine differences between participants in FAARM intervention and control groups using the aggregated pro-WEAI measures and each pro-WEAI domain and indicator separately.

## 2 Methods

We undertook this assessment using standard pro-WEAI tools (Malapit et al., 2019) among a sub-sample of participants in the FAARM cluster-randomized controlled trial (ClinicalTrials.gov ID: NCT025-05711), conducted in parts of 13 unions from Nabiganj and Baniachong sub-districts, Habiganj District, Sylhet Division, Bangladesh. The FAARM trial enrolled women whose self-reported age in 2014 was <30 years, who were interested in gardening, and had access to at least 40 m^2^ of land. Further information on the design is available in the FAARM study protocol (Wendt et al., 2019). FAARM aims to evaluate the impact of a homestead food production program implemented through the international non-governmental organization Helen Keller International.

### 2.1 FAARM intervention

Nutrition-sensitive agricultural programs, such as the homestead food production program implemented in the FAARM trial, have the potential to improve women's and children's nutrition and women's empowerment, which may synergistically lead to greater nutrition improvements (Hillenbrand, 2010, Kadiyala et al., 2014, Olney et al., 2016, Heckert et al., 2019, Quisumbing et al., 2020, Bushamuka et al., 2005, Baliki et al., 2019, Patalagsa et al., 2015). The FAARM intervention, undertaken over a roughly three-year period from mid-2015 to late 2018, consisted of forming groups of around 16 women (ranging from 8 to 26). Subsequently, 7-8 project staff trained these groups on nutrition and hygiene during courtyard sessions held approximately every two months (including sessions on childcare and feeding), and conducted training with hands-on demonstrations of improved gardening and poultry rearing practices. Approximately every other month, project staff conducted individual household visits with counseling to supplement the group training program by enabling targeted messages to be given to intervention participants as well as other family members. Selected women participants and their families received additional support to become group leaders. The intervention also distributed assets, such as seeds (seasonally) and small farm tools, and provided partial reimbursement for building a poultry shed.

The FAARM intervention did not include an explicit gender or empowerment curriculum, such as *Nurturing Connections* (Helen Keller International Bangladesh, 2015), though some activities from this curriculum were part of the courtyard sessions. Table 1 outlines key FAARM intervention activities and their potential impacts on the pro-WEAI indicators of agency. We hypothesized that household visits, followed by own productive activities and skill-building, would be the most consequential activities for building agency. Work balance was the only indicator that we hypothesized the intervention could make worse. Not directly pictured, reinforcing feedback loops were expected between agency domains. Prior qualitative work has investigated the pathway towards empowerment among women who participated in the FAARM intervention (Dupuis et al., 2022).

**Table 1:** Hypothesized impacts of key FAARM intervention activities on women's agency

	Skill building	Household visits	Group formation	Group leadership	Productive assets	Market access	Own productive activities
*Intrinsic agency*							
Intimate partner violence not acceptable		+	+	+			
Autonomy in income	+	+	+	+	+		+
Self-efficacy	+	+	+	+	+	+	+
Respect among household members	+	+		+	+		+

*Instrumental agency*							
Access to and decisions on financial services	+	+				+	+
Ownership of land and other assets		+			+	+	+
Input in productive decisions	+	+			+	+	+
Control over use of income	+	+			+	+	+
Visiting important locations		+	+	+		+	
Work balance	–	–/+	–	–		–	–

*Collective agency*							
Group membership	+	+	+	+			+
Membership in influential groups	+	+	+				+

The FAARM trial was undertaken in Habiganj District, Sylhet Division, Bangladesh.

While we identified the location for the FAARM trial primarily based on its high levels of undernutrition – after excluding wealthier areas and those with similar projects (Wendt et al., 2019), this part of Bangladesh is also notable for the limited role women have in society. In the 2014 Demographic and Health Survey (DHS), Sylhet was the division with the lowest proportion of women who were employed for cash income in the year before the survey, could visit a health facility alone or with a child, and participated in any of three household decisions: major household purchases, own health care, and children’s health care (National Institute of Population Research Training (NIPORT) et al., 2016). This pattern was largely the same in the 2017 DHS (National Institute of Population and Training (NIPORT) and ICF, 2020). However, women in this area still have predominant control over household garden management and products (Akhter et al., 2010). At baseline, women in our study setting had almost universally low freedom of movement, only infrequently met with other women in the community, and made few decisions apart from what to cook (Sinharoy et al., 2019, Sparling et al., 2020).

### 2.2 Study design

With this evaluation, we estimate the impact of FAARM’s HFP intervention on women’s empowerment four to five months after the end of the intervention. The counterfactual was the cluster-randomized control group. This assessment draws on data from two surveys: 1) the FAARM baseline and 2) the pro-WEAI. We administered the FAARM baseline survey to all women enrolled in FAARM from March to May 2015 and included questions on sociodemographic characteristics and other measures (Wendt et al., 2019). We conducted the pro-WEAI survey from April to May 2019.

#### 2.2.1 Pro-WEAI sampling and survey

The pro-WEAI survey tool was not available at the time of FAARM's baseline data collection. Moreover, as IFPRI was still developing the pro-WEAI at the time of the assessment, we could not undertake formal sample size calculations as effect size estimates were not available. IFPRI guidance recommended a sample of at least 300 wife-husband pairs. To balance the project's needs and capacity for data collection, we targeted five households in each of the 96 FAARM trial settlements for inclusion in the pro-WEAI survey, aiming for 480 wife-husband pairs.

To facilitate survey administration, we excluded households with more than ten members or more than one enrolled woman. This removed 20% of the enrolled women and somewhat reduced the proportion of women in the most educated category and the highest wealth tercile, as well as excluding some joint households, leaving more nuclear ones (Supplemental Table S1). From the list of eligible women, we selected women at random, first choosing from those we had previously chosen at random for inclusion in the food consumption surveillance (Wendt et al., 2019).

#### 2.2.2 Pro-WEAI training and data collection

The survey included all the modules of the pro-WEAI that were required in 2019 (Malapit et al., 2019) and was administered using tablets and the Open Data Kit (ODK) platform (Hartung et al., 2010).^2^ IFPRI provided a translation of most sections of the standard survey from English to Bengali, which was revised and contextualized to the local study area by an experienced team at the Helen Keller International – Bangladesh Country Office (translation available upon request). For data collection activities, we employed different staff who had a separate management structure from the FAARM implementation team. For the pro-WEAI survey, eight data collectors (four male and four female) were trained for seven days, including two practice field visits in the study area. Data collectors worked in set teams of one man and one woman, except in cases of illness. Data collectors administered the questionnaire only to respondents of their own sex.

The response rate was high, with 457 out of the targeted 480 women (95%) and 428 out of the targeted 480 men (89%) interviewed. The proportion of the sample reached was slightly greater in intervention areas compared to control areas (women: 96% vs. 95%; men: 91% vs. 87%). In total, paired surveys from 420 couples were obtained (87%), with attainment again slightly higher in the intervention group than in the control (90% vs. 85%).

### 2.3 Data management and analysis

Data were processed and analyzed in Stata 15.1 (Statacorp 2017). After initial data processing, we adapted and applied standard pro-WEAI Stata *.do files from IFPRI. The *.do files calculated the three standard pro-WEAI scores as well as Table 3 and Fig. 2. For use in background tables and regression analysis, we extracted relevant indicators from the FAARM baseline dataset. We calculated household wealth terciles using standard techniques, including weighting by household size, for the FAARM study population as a whole (Rutstein & Johnson, 2004). We created four mutually exclusive groups based on years of education received: 1) No education, 2) Partial primary: 1 to 4 years of schooling, 3) Complete primary: exactly five years of schooling, 4) Any secondary: more than five years of schooling. We calculated years since marriage at baseline by subtracting the woman's reported age at marriage from her age at baseline. For the one couple for whom the woman's age at marriage was not reported, we imputed this value using the woman's current reported age. We defined nuclear households as a household with no more than one ever-married woman and one ever-married man, and thus households with one married couple and an elderly parent were counted as joint. All other variables, except the pro-WEAI indicators, were used directly as asked in the questionnaire.

**Table 2:** Household and individual characteristics of respondents, by sex of respondent and FAARM intervention group.

	Women		Men	
	Control	Intervention	Control	Intervention
	% or mean	% or mean	% or mean	% or mean
*Education*^*1*^				
No education	19%	19%	37%	37%
Partial primary	22%	23%	19%	18%
Complete primary	24%	18%	17%	19%
Any secondary education	35%	40%	27%	26%
				
*Household wealth tercile^1#^*				
Lower	44%	41%	45%	42%
Middle	35%	37%	35%	37%
Upper	20%	23%	19%	21%
				
*Other household characteristics*				
Hindu^1^	29%	27%	31%	26%
Nuclear family at baseline^1^	40%	44%	40%	43%
Nuclear family at pro-WEAI^2^	59%	55%	59%	54%
				
*Mean of continuous variables*				
Age in years^1^	25.0	24.9	33.1	33.4
Years since marriage^1^	7.0	7.2	6.9	7.1
Household members^2^	5.4	5.8	5.4	5.9
				
Decisions made by women*^1^				
Food preparation	72%	78%		
Major purchases	28%	29%		
Daily purchases	55%	52%		
Own healthcare	26%	25%		
				
Mobility (last month)*^1^				
Market	4%	3%		
Health facility	8%	7%		
Community meeting	2%	0%		
Relative's or friend's house	23%	19%		

The FAARM trial was undertaken in Habiganj District, Sylhet Division, Bangladesh. n = 885 except for where *n = 883 due to missing information.

# Wealth terciles are constructed for the FAARM study population as a whole and weighted by household size.

1 Data source: FAARM baseline survey (March–May 2015).

2 Data source: pro-WEAI survey (April–May 2019).

**Fig. 1:**
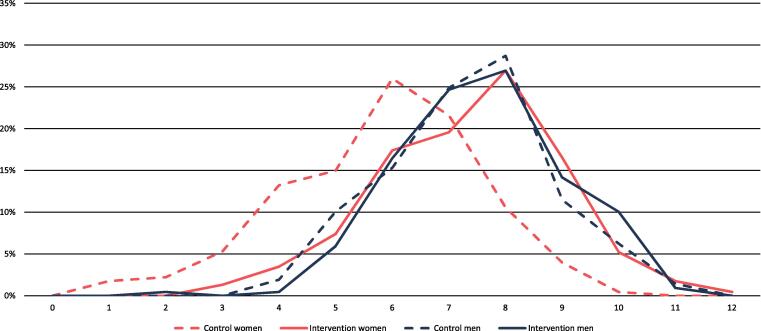
Density plot of the number of indicators for which empowerment was attained, by sex and FAARM intervention group. The proportion of respondents, by sex and intervention, who achieved empowerment on the number of indicators displayed on the x-axis. Blue is for men and red for women, while the dashed lines are for control group and the solid lines for the intervention group. The vertical green line corresponds to the 0.75 empowerment cutoff on the 3DE scale. The area under the curve to the right of this line corresponds to the proportion of respondents defined as empowered in agency by the pro-WEAI. The FAARM trial was undertaken Habiganj District, Sylhet Division, Bangladesh. n = 885. (For interpretation of the references to color in this figure legend, the reader is referred to the web version of this article.)

**Fig. 2:**
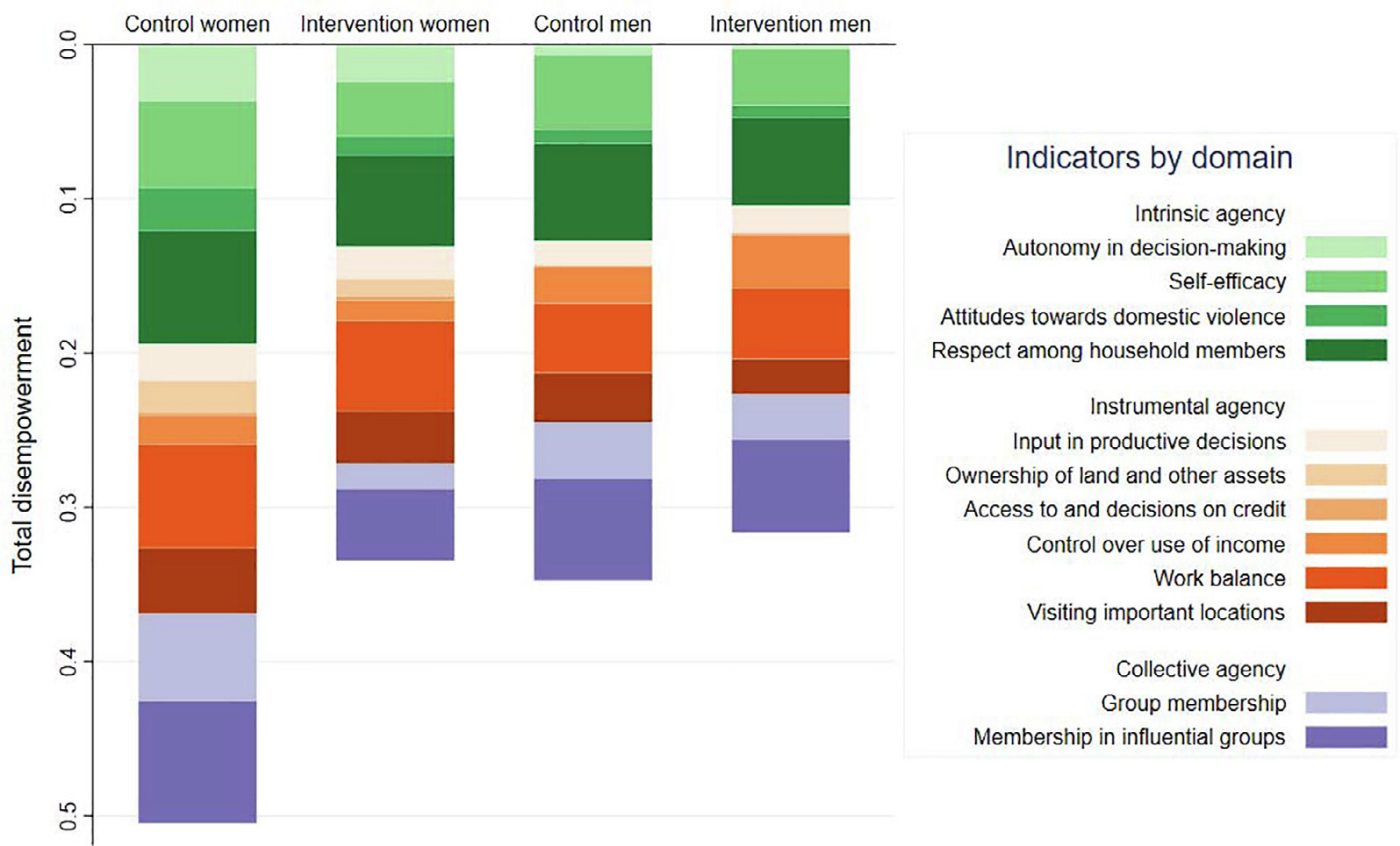
The indicator- and domain-wise contributions to disempowerment in agency, by sex and FAARM intervention group. For men and women who were disempowered, the figure depicts the absolute contribution of each indicator to disempowerment in agency for men and women. Each bar's depth shows the total disempowerment (1- 3DE), and different colors within show the absolute contribution of each indicator to disempowerment in agency. Indicators are color-coded by domain with shades of green for intrinsic agency, orange for instrumental agency, and purple for collective agency. The FAARM trial was undertaken in Habiganj District, Sylhet Division, Bangladesh. (For interpretation of the references to color in this figure legend, the reader is referred to the web version of this article.)

#### 2.3.1 Statistical methods

Analysis of the pro-WEAI provides three scores: 1) the three domains of empowerment score (3DE), calculated separately for men and women; 2) the gender parity index (GPI), calculated on the couple level; and 3) the pro-WEAI score, calculated by taking a weighted average of the 3DE, with a weight of 90%, and the GPI, with a weight of 10%. In our study, we calculated all scores separately by intervention and control group.

As the three pro-WEAI scores are single numbers constructed on the population level, statistical comparisons are not possible on the scores themselves. To study the FAARM intervention's impact on women's agency as measured through the pro-WEAI, we used the individual-level indicators generated during 3DE score construction before averaging over the population. We also present the variables that underlie these indicators, descriptively as means and proportions, to identify those responsible for any differences in the indicator's value. We did not undertake statistical tests on these underlying variables as the sheer number of comparisons would make interpretation difficult.

To estimate the FAARM intervention's impact on the pro-WEAI indicators, we used multilevel logistic and linear regression models, for dichotomous and continuous outcome indicators respectively, with random effects on the data collector and settlement (cluster) levels, with and without controls for education, religion, wealth, age, years since marriage, household size, and family structure. We included these person-level covariates to compare men and women in the same model, as differences in these covariates between sexes could cause the observed differences in their agency. Including these and household-level covariates in the model also helps to more precisely estimate FAARM's impacts by reducing sources of variation. The impact was estimated using an interaction term between the sex of the respondent and intervention status to compare the intervention's impact on men and women explicitly. We undertook a sensitivity analysis using a three-level structure with random effects on the data collector, settlement (cluster), and household levels, with similar results for indicators for which the model would converge (available on request).

Due to the large number of clusters randomized in the FAARM trial (Wendt et al., 2019), a single post-intervention assessment of empowerment was sufficient to estimate program impact (Glennerster and Takavarasha, 2014). These clusters were allocated to intervention and control arms using covariate-constrained randomization after cluster and individual recruitment had been completed (Bolzern et al., 2019, Hayes and Moulton, 2017). We observed good balance in these underlying characteristics of the population (Table 2).

### 2.4 Ethics

The FAARM protocol was positively reviewed by the ethics committees in Bangladesh and Germany (Wendt et al., 2019). We obtained written informed consent from all study participants at baseline.

## 3 Results

Household and individual characteristics of the surveyed population are shown in Table 2, disaggregated by the respondent's intervention group and sex. Husbands were on average eight years older than their wives. Education levels were low, and women had received more education than men. Across groups, between 26 and 31% of the population was Hindu, with the remainder Muslim, the majority religious group in Bangladesh. Around 40% of respondents resided in a nuclear family at baseline, increasing to around 55% by the end of the intervention. The average household had 5.4 members – larger than the national average of 4.5 (National Institute of Population Research Training (NIPORT) et al., 2016). Couples had been married, on average, seven years before the baseline survey in 2015.

The lower half of Table 2 provides estimates for selected indicators of empowerment available in the baseline survey. While around three-fourths of women were involved in decisions regarding food preparation, only a little over half took part in decisions about daily purchases, and only around a quarter were involved in decisions involving their own healthcare and major household purchases. Women only infrequently left their homesteads, with only one in five visiting relatives’ or friends’ houses in the last month, the most frequently listed location. There are no notable differences between the intervention and control groups.

### 3.1 Description of pro-WEAI scores

There are twelve individual indicators of agency that underlie the pro-WEAI, each with its own empowerment condition (further detailed in the supplementary material). The distribution of the number of pro-WEAI indicators for which empowerment was attained was similar between women in the intervention and men in both groups. In contrast, the distribution of scores for women in the control group was separate and to the left of the other three groups, corresponding to lower empowerment. The peak of the distribution for men and intervention group women was eight, as compared to six for control group women (Fig. 1).

In line with this, all three standard pro-WEAI scores were higher in the intervention than in the control group (Table 3, figures in bold). Differences in the 3DE score, or the average percentage of indicators for which respondents attained empowerment, were negligible between men and women in the intervention group (0.02) but larger in the control group (0.15). Corresponding to this, the difference in 3DE score between intervention and control groups was greater for women (0.16) than men (0.03). The proportion of each population considered empowered in agency, i.e., the proportion that obtained empowerment on nine or more indicators (3DE score equal to or greater than 0.75), also reflects the larger 3DE scores. Roughly one-quarter of men and women in the intervention group were empowered in agency compared to only 4% of women and 19% of men in the control group.

**Table 3:** Pro-WEAI scores, by sex of respondent and FAARM intervention group.

	Control		Intervention		Difference	
Indicator	Women	Men	Women	Men	Women	Men
Number of observations	227	209	230	219		
**Three domains of empowerment (3DE) score**	**0.50**	**0.65**	**0.66**	**0.68**	**0.16**	**0.03**
% empowered	4%	19%	24%	25%	20%	6%
Mean 3DE score for not yet empowered	0.47	0.57	0.56	0.58	0.09	0.01
Number of dual-adult households	205		215			
% with gender parity	29%		54%		25%	
Average agency gap	0.34		0.26		−0.08	
**Gender Parity Index (GPI)**	**0.76**		**0.88**		**0.12**	
**Pro-WEAI score**	**0.52**		**0.69**		**0.17**	

The FAARM trial was undertaken in Habiganj District, Sylhet Division, Bangladesh. n = 885.

Among the individuals who did not meet the empowerment threshold (3DE score < 0.75), the mean 3DE score was higher for the intervention group (both sexes) and men in the control group, all approximately 0.57, compared to women in the control group (0.47). For these disempowered individuals, differences in intrinsic agency and collective agency were large contributors to the greater disempowerment among women in the control group compared to women in the intervention group (Fig. 2).

Gender parity between couples was greater in the intervention group (Table 3), with a majority (54%) of women empowered (equal to or greater than 0.75 on the 3DE scale) or at least as empowered as their male partner, compared to only 29% of women in the control group. In addition, the average gap between husbands' and wives' 3DE scores in households with disempowered women was slightly lower in the intervention group, with a 0.26 gap, compared to the control group, with a 0.34 gap. These two measures, gender parity and the gender parity gap, combine to make the gender parity index (GPI). The GPI was higher in the intervention group (0.88) than in the control group (0.76). The pro-WEAI score is calculated from the women's 3DE and GPI. The overall pro-WEAI score was higher in the intervention group (0.69) than in the control group (0.52), indicating a higher and more equitable distribution of agency.

### 3.2 Indicator-level analysis

**Fig. 3:**
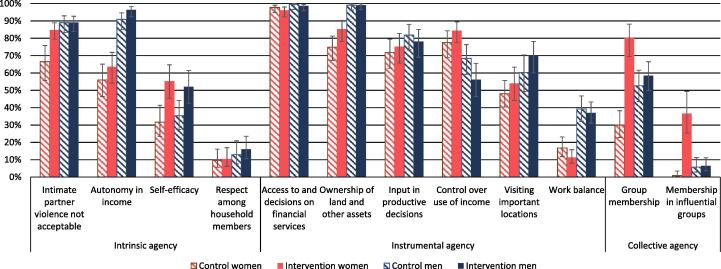
Empowerment on each 3DE indicator, by sex and FAARM intervention group. The proportion of respondents, by sex and intervention group, categorized as empowered on each indicator. Blue is for men and red for women, while the hatched bars are for the control group and the solid bars for the intervention group. The FAARM trial was undertaken in Habiganj District, Sylhet Division, Bangladesh. n = 885. (For interpretation of the references to color in this figure legend, the reader is referred to the web version of this article.)

Attainment of empowerment varied greatly over the twelve individual indicators that go into the 3DE score (Fig. 3 and Supplemental Table S2), ranging from near-universal empowerment on “Access to and decisions on financial services” to less than one-fifth of any group obtaining empowerment on “Respect among household members.” In this section, we examine how attainment on each indicator varies across sex and intervention groups, and which elements of each indicator account for the variation (Supplemental Tables S3–S9 and Supplemental Figs. S1–S7). Furthermore, we estimate intervention effects using multivariable logistic and linear regression (Table 4 and Supplemental Table S12).

**Table 4:** Impact of the FAARM intervention on pro-WEAI indicators, by sex of respondent.

Categorical measures				
	Women		Men	
	Odds ratio	p-value	Odds ratio	p-value
*Intrinsic agency*				
Intimate partner violence not acceptable	3.5	<0.001	0.9	0.864
Autonomy in income	1.7	0.045	2.6	0.047
Self-efficacy	3.2	<0.001	2.3	0.002
Respect among household members	1.0	0.937	1.3	0.462

*Instrumental agency*				
Access to and decisions on financial services	0.7	0.623	0.5	0.551
Ownership of land and other assets	2.6	0.001	1.0	0.960
Input in productive decisions	1.2	0.549	1.0	0.881
Control over use of income	1.8	0.042	0.7	0.118
Visiting important locations	1.1	0.615	1.7	0.052
Work balance	0.7	0.153	1.0	0.834

*Collective agency*				
Group membership	14.0	<0.001	1.5	0.117
Membership in influential groups	166.8	<0.001	1.4	0.557
Empowered in agency	7.7	<0.001	1.5	0.160
Women's equity with spouse*	3.5	<0.001		

Continuous measures				

	Women		Men	
	Coefficient	p-value	Coefficient	p-value

3DE score	1.51	<0.001	0.30	0.036
Intrinsic agency	0.46	<0.001	0.22	0.005
Instrumental agency	0.18	0.068	-0.01	0.949
Collective agency	0.86	<0.001	0.09	0.172

Results are based on multilevel logistic and linear regression models with random effects on the data collector and cluster levels. Model includes sex, education, religion, wealth, age, years since marriage, household size, and family structure. The results of the models without covariates are given in Supplemental Table S10 while full regressions results with coefficients for all covariates are given in Supplemental Table S12. The FAARM trial was undertaken in Habiganj District, Sylhet Division, Bangladesh. n = 885 except for “Women's equity with spouse” where *n = 420.

#### 3.2.1 Intrinsic agency

Among the four intrinsic agency indicators, empowerment in finding intimate partner violence unacceptable was highest overall but substantially lower among control group women than among men or intervention women (Fig. 3 and Supplemental Table S2). These differences are linked to control group women more commonly stating that violence was acceptable under circumstances such as going out without informing, neglecting the children, or arguing (Supplemental Table S3). For instance, 23% of women in control areas agreed with the statement that violence against women was acceptable for arguing, compared to only 10% of women in the intervention group and 4–5% of men. There was strong evidence for a difference also from multivariable logistic regression, with the odds of women being empowered on this indicator three times higher (OR 3.5, p < 0.001) in the intervention group compared to control, with no difference among men (Table 4).

Empowerment concerning autonomy in income varied considerably between the sexes, but less between intervention and control: while over 90% of men were empowered on this indicator (intervention 96%; control 91%), only a little over half of the women were (intervention 63%; control 56%). This difference was due to fewer women who reported using income as they personally wanted to (Supplemental Table S4), the only variable of the four that were collected that went into the final indicator. Many people reported that they did not have alternatives to spending their money (Supplemental Table S4). There was weak evidence of a positive impact of the FAARM intervention on the income autonomy indicator among men (OR 2.6, p = 0.05) and women (OR 1.7, p = 0.05, Table 4).

In contrast, attaining self-efficacy did not differ much between sexes but was considerably higher in the intervention group (men 52%; women 55%) than in the control group (men 35%; women 32%). Individuals in the intervention group scored higher across all variables that comprise this indicator (Supplemental Fig. S1), e.g., 19% more women and 11% more men reported agreeing or strongly agreeing that they perform very well in tough times. There was strong evidence of a sizable impact of the FAARM intervention on this indicator (Table 4), with over double the odds of adequate self-efficacy in the intervention group compared to control among women (OR 3.2, p < 0.001) and men (OR 2.3, p = 0.002).

Being empowered concerning respect among family members was less common in our study population, ranging from 10% among women in both groups to 13% among men in the control group and 16% among men in the intervention group. Among the variables that make up this indicator, men reported showing respect for (and receiving respect from) their spouses more frequently than women did (Supplemental Fig. S2). Over one-third of men reported feeling comfortable disagreeing with their spouse compared to only one-fifth of women. Despite the subtly greater levels of respect seen in the intervention areas across most underlying variables (Supplemental Fig. S2), there was no evidence that the FAARM intervention impacted this indicator (Table 4).

#### 3.2.2 Instrumental agency

Empowerment concerning access to and decisions on financial services was nearly universal though a little lower for women compared to men (Fig. 3 and Supplemental Table S2). Loan services were available in nearly all areas, and, for most loan types, both husbands and wives decided to take the loan, indicating a joint decision, and only slightly less often how to use the loan (Supplemental Table S5). There was no evidence of the FAARM intervention impacting this indicator (Table 4).

Ownership of land and other assets was nearly universal for men (99%), while only 75% of control women and 85% of intervention women were empowered on this indicator (Fig. 3 and Supplemental Table S2). For household asset ownership, the only large difference was that intervention households were more likely to own poultry – 35% without any ownership in control, compared to 22% in intervention areas (Supplemental Table S6). Within households, men owned assets more than women did, with small consumer durables and poultry being the only exceptions. Nearly half of intervention women, 44%, owned non-mechanized farm equipment (97% of households minus 53% women not owning) compared to only 12% of women in the control group (94% of households minus 82% of women not owning; Supplemental Table S6). In the aggregated indicator, differences in the number of assets owned between intervention and control groups were relatively small, compared to the much larger difference between men and women (Supplemental Fig. S3). There was strong evidence that intervention women had higher odds of empowerment on this indicator than control women (OR 2.6, p = 0.001, Table 4).

The proportion of the population with input into productive decisions did not vary substantially by sex or intervention group (Fig. 3 and Supplemental Table S2), although there were some differences in the underlying variables that went into this indicator (Supplemental Table S7). Participation in horticulture and poultry was similar among men and women and higher in intervention than control areas (96% vs. 81%) (Supplemental Table S7). However, the control that women had over horticulture and poultry was similar between the intervention groups. In contrast, a lower proportion of women than men participated in fish-pond activities, and even if they participated, most had lower input into decisions than men. There was no evidence of the FAARM intervention impacting this indicator (Table 4).

In contrast to most other indicators, control over the use of income from on-farm and off-farm activities was higher in women (control 78%; intervention 84%) than men (control 68%; intervention 56%, Fig. 3 and Supplemental Table S2). This indicator includes both decisions about income and about the consumption of products from the listed activities. While men participated in horticulture and poultry production, they had less input into the products' use (Supplemental Fig. S4). For off-farm activities, the reverse was seen, with men having a greater say in decisions (Supplemental Fig. S5). There was weak evidence for an impact of the FAARM intervention on increasing empowerment on this indicator among women (OR 1.8, p = 0.04) and no evidence for an impact among men (Table 4).

Empowerment in visiting important locations, or mobility, was higher for men (control 60%; intervention 70%) than for women (control 48%; intervention 54%, Fig. 3 and Supplemental Table S2). Women visited markets and urban centers much less frequently than men, while men visited health centers less frequently (Supplemental Fig. S6). Women in intervention settlements went to meetings more often than women in control settlements. There was weak evidence of an impact of the FAARM intervention on this indicator among men (OR 1.7, p = 0.05, Table 4) but none among women.

Empowerment in work balance, defined in the pro-WEAI as working <10.5 h daily (with childcare as secondary activity counting at half the time spent), was low overall, but higher among men, at over one-third, and lower among women, less than one-fifth (Fig. 3 and Supplemental Table S2). The absolute amount of time spent on work and non-work primary activities was similar between men and women (Supplemental Table S8), so the difference in empowerment was mostly related to childcare as a secondary activity. There was no evidence of the FAARM intervention impacting this indicator positively or negatively (Table 4). The time spent on work (9.3 h) vs. non-work activities (14.7 h) per day was identical for intervention and control women, with poultry and horticulture taking up only 19 min on average for intervention women compared to 11 min for control women (p = 0.31).

#### 3.2.3 Collective agency

A little over half of men were considered empowered in terms of group membership, while women's results varied by intervention arm, with 80% attaining empowerment in the intervention group and 30% in the control group (Fig. 3 and Supplemental Table S2). Men were more often members of all types of groups, except microfinance groups and producer groups in intervention areas (Supplemental Table S9). The FAARM intervention resulted in much higher odds of empowerment on this indicator among women (OR 14.0, p < 0.001) with no impact among men (Table 4).

Only 6% of men attained empowerment as a member of an influential group, compared to 1% of women in the control group and 37% of women in the intervention group (Fig. 3 and Supplemental Table S2). Only a few group members perceived the groups to which they belonged as influential (Supplemental Fig. S7), except for the water users' and insurance groups in which few people were members (Supplemental Table S9). In contrast, around half of the women who belonged to producer groups in the intervention area considered their group influential (Supplemental Fig. S7). The FAARM intervention thus resulted in a huge impact on this indicator among women (OR 167, p < 0.001) with no impact among men (Table 4).

### 3.3 Analysis of summary measures

Although the proportion of individuals classified as empowered using the 3DE score was greater in the intervention group compared to control (Table 3), there was only evidence for an impact of the FAARM intervention on women's agency (OR 7.7, p < 0.001, Table 4) and no evidence for an impact among men (OR 1.5, p = 0.16). In terms of continuous measures, intervention group women had higher average 3DE scores (Fig. 1 and Table 3), driven by higher scores in all three domains of intrinsic, instrumental, and collective agency (Supplemental Fig. S8, and Supplemental Table S11). The FAARM intervention improved agency scores for both men and women overall, with strong evidence for a large impact on women's scores (empowerment on 1.5 more indicators than control, p < 0.001) and weaker evidence for an impact on men’s scores (empowerment on 0.3 more indicators, p = 0.04, Table 4). FAARM increased women's scores through all domains but with weak evidence (p = 0.07) for an improvement in instrumental agency (Table 4). Among men, FAARM improved empowerment scores only through intrinsic agency (Table 4). In line with this, gender equity was higher in the FAARM intervention group than in the control group (OR 3.5, p < 0.001, Table 4). Full regression results are available in Supplemental Table S12.

## 4 Discussion

In one of the first studies using the pro-WEAI (Quisumbing et al., 2020) in a randomized controlled trial, we found that a homestead food production program increased women's agency related to agriculture in rural Sylhet, Bangladesh, bringing it up to levels similar to men. We saw improvements in all three indicator domains measured by the pro-WEAI, with gains primarily in women's intrinsic and collective agency. For men, there was a smaller improvement in intrinsic agency. These changes are notable because – while providing opportunities and resources for empowerment – the activities encouraged by the FAARM intervention fit into women’s traditional gendered roles.

### 4.1 Intrinsic agency

Overall, higher intrinsic agency for women was driven by increases in finding intimate partner violence not acceptable, autonomy in income, and self-efficacy. Although the FAARM intervention did not explicitly discuss intimate partner violence, increased social support via group membership and household visits by program facilitators and group leaders could be potential pathways (Table 1). In line with these findings, a previous study in Bangladesh found a sustained reduction in experienced intimate partner violence after an intervention that included cash transfers and nutrition behavior change communication, also delivered via group sessions and household visits, compared to cash transfers alone (Roy et al., 2019). In contrast, the evaluation of a more recent program in Bangladesh with more explicit gender components found no impact on intimate partner violence (Quisumbing et al., 2020).

Demographic and Health Surveys (DHS) have included a module on acceptance of intimate partner violence for many years, the same as used in the pro-WEAI. Cognitive testing of this module as part of the pro-WEAI in Myanmar raised potential issues with understanding due to translation difficulties and respondents wanting further clarification on the hypothetical scenarios presented (e.g., what was the intention behind leaving without informing) (Lambrecht et al., 2020). In contrast, Yount et al., in an analysis of pro-WEAI measurement properties using data from Bangladesh and Burkina Faso, found that this module was the only one tested that met all validation assumptions (Yount et al., 2019).

Concerning autonomy in income, more men and women in our intervention group reported that they spent income in a way they personally felt was right, compared to men and women in the control group. Skill building and practice of new skills as part of the FAARM intervention may have led to higher prioritization of nutrition and health and thus to higher confidence in asset use and spending in ways that would lead to better health outcomes (Table 1). However, according to feedback from our data collection team, respondents struggled to answer these questions, which were presented as hypothetical scenarios (e.g., describing how someone spent his/her income and then asking “are you like this person?”). The final indicator was based only on the question about being like the person who “used income as they personally felt was right.” During cognitive testing in Myanmar, respondents reported challenges in generalizing this indicator's question set to their personal experiences (Lambrecht et al., 2020). Further, statistical analysis of pro-WEAI data from Bangladesh of a previous and longer version of the income use variables indicated that this set could not be psychometrically validated due to violations of model assumptions, poor model fit, and a lack of a strong relationship with the underlying agency construct (Yount et al., 2019). Therefore, results for this indicator should be interpreted with caution.

Several components of the FAARM intervention may have worked synergistically to achieve a substantial improvement in self-efficacy among both men and women. Women participated in training to build their skills in gardening, poultry rearing, nutrition, and child care. They received productive assets, individualized support via household visits, and social support through group membership to foster the adoption and continued practice of homestead food production to better feed themselves and their families. For the approximately 10% of women who became group leaders, the extra training and opportunity to support and teach their fellow group members may have further increased their self-efficacy (Table 1). In Nepal, a qualitative study of a Helen Keller International homestead food production program also found improvements in self-efficacy (Kjeldsberg et al., 2018). The pro-WEAI measures self-efficacy using the well-validated New General Self-Efficacy scale, increasing confidence in these results (Chen et al., 2001).

While the intervention could theoretically improve respect among household members indirectly through several pathways (Table 1), there was no curriculum component specifically addressing this topic. The particular set of questions on respect within the household also presented issues in cognitive testing studies, revealing little variation in Myanmar, perhaps due to the importance of respect in these communities, leading to high levels throughout (Lambrecht et al., 2020). A cognitive testing study in Bangladesh reported that many respondents interpreted “respect” in the sense of “honor.” Though these are similar, the authors recommended careful discussion and examples to ensure similar understanding across interviewers and respondents (Hannan et al., 2020). In light of these recommendations, our translation of this module was revised during field testing. Yount et al. did not assess this indicator's validity as they did not consider it a measure of agency (Yount et al., 2019).

### 4.2 Instrumental agency

Overall, there was less change in instrumental agency between intervention and control groups compared to the other domains. We found no impact of the FAARM intervention on “access to and decisions on financial services,” likely due to the very high existing levels of microfinance coverage and use in our project area. We also did not find an intervention impact on input into productive decisions, although intervention areas did have higher participation in horticulture and poultry, similar to increases seen in earlier studies (Patalagsa et al., 2015).

More women in the intervention group reported owning “land and other assets.” In particular, more intervention women owned non-mechanized farm equipment, likely assets distributed through FAARM, e.g., poultry sheds (intervention-subsidized), watering cans, shovels, and feeding pots for poultry. As we conducted the pro-WEAI survey several years after assets had been distributed, this indicates that ownership of these assets remained at least partially with the women. Intervention households were also more likely to own poultry, a practice encouraged and supported by FAARM. Homestead food production also supports the cultural norm of women controlling income and outputs from horticulture and poultry in Bangladesh, leading to a greater level of control over the use of income among intervention women.

FAARM’s impact on women’s control over the use of income did not appear to be driven by any particular on-farm or off-farm activity. This is not too surprising as nearly all women, both in intervention and control areas, who were participating in poultry and horticulture – the primary activities promoted by FAARM – had at least some decision-making power over these activities. In contrast, fewer men had decision-making power over the direct consumption of these commodities, resulting in empowerment on this indicator being higher for women than men. Homestead activities, such as poultry rearing and horticulture, are usually seen as women’s domains in Bangladesh (Patalagsa et al., 2015, Akhter et al., 2010), and this is in line with the design of the homestead food production programs to support women “[to] enhance their bargaining power and become more productive in their traditional roles” (Iannotti et al., 2009), rather than directly confronting gender inequality.

No impact was seen on mobility, as measured by visiting important locations. However, intervention women reported going to meetings more often than control women. As the intervention had concluded at least four months before the survey, these continued meetings speak to self-sustaining groups in some areas, confirmed by qualitative interviews as part of a related study (Dupuis et al., 2022). While it is conceivable that the FAARM intervention could increase mobility through improved market access (Table 1), the lack of impact seen is not unexpected, given that women's mobility in Sylhet is highly restricted (Sinharoy et al., 2018, National Institute of Population Research Training (NIPORT), Mitra Associates, & ICF International, 2016). More fundamentally, it is debatable whether visiting frequency should be a component of instrumental agency (Yount et al., 2019), as not having visited certain places does not necessarily signify the inability to go, and at the same time, having visited, for example, a health facility, does not imply a desire, but could be out of sheer necessity.

We also saw very little change in workload among both men and women. This is reassuring given that an increased workload for women could be a potential unintended consequence of this type of program, e.g., when women add gardening, poultry rearing, and product sales to usual child care and domestic work (McGuire and Popkin, 1990, Kjeldsberg et al., 2018). The additional amount of time per day that women spent on horticulture activities in the intervention area (5 min) is in line with previous studies in Bangladesh (6 min) (Patalagsa et al., 2015).

### 4.3 Collective agency

Women in the intervention arm of the FAARM trial were much more likely to be a member of a group – a consequence of the intervention forming producer groups and participants becoming members – and they were more likely to perceive the group as influential in the community. These findings are notable as the survey took place four months after the intervention finished, indicating that many groups continued to exist. We posit that skill-building, the practice of learned skills, and household visits to support these activities motivated the women to maintain group membership and recognize their groups as influential (Table 1). Researchers also found that homestead food production programs in Burkina Faso and Bangladesh increased women's social interactions with each other (Olney et al., 2016, Patalagsa et al., 2015).

The psychometric validation of the pro-WEAI by Yount et al. did not include the collective agency module. They highlight that collective agency in the pro-WEAI is based exclusively on the two questions on group membership, excluding other potentially influential pathways such as non-institutional collective action including community projects or social support among other families in the community (Yount et al., 2019). A further issue is that the initial question asks if a group is “in your community,” thereby excluding relevant groups that may be operating outside of the respondent's community (Lambrecht et al., 2020). This exclusion is likely more problematic for men's responses due to women's mobility restrictions in our project setting.

### 4.4 Overall empowerment and equity

Overall, there was strong evidence that FAARM substantially improved women's agency and thus increased equity between spouses. The ANGeL project, a multi-arm trial in Bangladesh that evaluated nutrition, agriculture, and gender interventions, also part of the GAAP2 portfolio, reported somewhat smaller increases in women's empowerment and comparable improvements in men's empowerment (Quisumbing et al., 2021). The FAARM intervention improved women's intrinsic and collective agency, with smaller improvements in instrumental agency. Overall empowerment was also improved for men, but to a much smaller extent and only in the intrinsic agency domain. This impact aligns with the FAARM intervention's aims to focus on and support women through group and individual sessions, fostering best practices in home gardening and poultry rearing, typical female responsibilities in Bangladesh. Men and other household members were brought into the intervention primarily through household visits and invitations to selected group sessions. In many households, family members did participate and support, particularly in group leader households and for fertilizer preparation (Dupuis et al., 2022).

### 4.5 Strengths and limitations

This study has several strengths. It is one of the first to use the pro-WEAI modules to assess the impact of a nutrition-sensitive agriculture intervention on women's agency, using a cluster-randomized controlled trial design. As one of thirteen programs in the GAAP2 portfolio, our results will be compared with other participating trials in upcoming publications and future studies that utilize the pro-WEAI tools. Furthermore, the pro-WEAI complements other data collected through FAARM, further enabling us to examine program impact pathways through agency.

This study's limitations include some respondents' difficulties with questions covering hypothetical scenarios or abstract concepts, as well as social desirability bias that may have affected responses. High interviewer skill was required to administer this survey. Difficulties in understanding questions have been reported in other studies cognitively testing the pro-WEAI modules (Lambrecht et al., 2020, Hannan et al., 2020). We cannot rule out uneven quality and low validity of some responses, particularly for the modules covering autonomy in income and respect among household members. To partially account for this, we included data collectors as random effects in our regression analyses. Another limitation, due to the way the indicators were defined, is that in some instances we did not see a change in an indicator despite improvements in underlying variables. For example, we saw clear increases in horticulture and poultry participation, and almost all participating women reported at least moderate input, but we found no impact on the “input into productive decisions” indicator as it only considers input among those who participated, excluding women who reported no participation, and thereby this indicator did not capture increases in participation.

Another limitation is that we are limited to a single post-intervention assessment of empowerment. Though this is sufficient to estimate program impact, we cannot know if these differences are due to improved agency in the intervention group, or the intervention protecting participants from secular reductions in empowerment. We argue that the former seems more likely, given the national improvements seen in gender indicators in recent years (World Economic Forum, 2021). Furthermore, the lack of a pre-intervention assessment limits our ability to estimate the level of baseline agency at which such effects may be possible, i.e., lower levels of baseline empowerment may mean greater potential for improvement.

Overall, the pro-WEAI comprises a comprehensive array of theory-driven indicators and data collection instruments that can be used to gather in-depth data in a comparable way across contexts. Nevertheless, both studies that conducted cognitive interviewing of pro-WEAI measures (Hannan et al., 2020, Lambrecht et al., 2020) highlighted outstanding issues. A study using pro-WEAI data from Bangladesh could not psychometrically validate the survey instrument (Yount et al., 2019). Some modules within the survey, such as those on intimate partner violence and self-efficacy, come from externally validated instruments (Yount et al., 2019). However, the modules newly developed for the pro-WEAI are not yet validated. Therefore, results should be treated with caution. Further refinements of the pro-WEAI instrument to add nuance and understandability, eliminating item sets that measure similar constructs, and streamlining the questionnaire to improve respondent focus were recommended (Yount et al., 2019).

### 4.6 Implications and next steps

Our findings support some but not all the hypothesized pathways of FAARM’s impact on women's agency (Table 1). The ability of nutrition-sensitive agriculture interventions to positively impact women's empowerment has often been hypothesized (Kadiyala et al., 2014, Ruel et al., 2018), but few studies have documented this in the context of a randomized controlled trial (Olney et al., 2016, Heckert et al., 2019, Quisumbing et al., 2020). We found strong evidence for improvements in agency overall as well as in specific domains and indicators. Additionally, we did not find any detrimental impacts of the program, such as increased workload for women. To interpret the magnitude of the differences found, it will be critical to compare our results to other programs across contexts. Our survey took place 4–5 months after intervention completion, indicating a continued impact on agency in the short term. Future rounds of data collection would be needed to show whether these impacts were sustained over a longer period.

The pro-WEAI includes a comprehensive array of indicators making up three domains of women's agency. However, many other aspects of empowerment exist. The pro-WEAI protocols, recognizing this, also include a recommended qualitative protocol to understand the local conceptualization of women's empowerment. Future qualitative analyses include understanding other aspects of FAARM intervention’s impact as described by participants and conducting a more in-depth assessment of self-efficacy in the FAARM population. Building on these pro-WEAI impacts, we also plan to measure how increased agency in agriculture may have improved nutritional outcomes using mediation analysis. Moving beyond pro-WEAI indicators, we plan to examine aspects of empowerment that are not tied to agriculture or agency, such as improvements in skills, decision-making power, and household recognition, which have been documented in other settings (Olney et al., 2016, Kjeldsberg et al., 2018).

## 5 Funding disclosure

This manuscript builds upon work undertaken as part of two research projects: 1) Gender, Agriculture, and Assets Project Phase Two (GAAP2) at the International Food Policy Research Institute (IFPRI) and 2) Food and Agricultural Approaches to Reducing Malnutrition (FAARM), based at Heidelberg University until 2019 and now at Charité – Universitätsmedizin Berlin. The German Ministry of Education and Research (BMBF) (grant number: 01ER1201) is the primary funder for the FAARM trial. FAARM's GAAP2 work was financially supported by a grant from IFPRI to Heidelberg University [IFPRI Project No. 301005.001.002.520.01.01]. The Bill & Melinda Gates Foundation (BMGF) [Grant number: INV-008977], the United States Agency for International Development (USAID) [Grant number: EEM-G-00–04-00013–00], and the CGIAR research program on Agriculture for Nutrition and Health jointly support IFPRI's GAAP2 work.

The content of this publication is solely the responsibility of the authors and does not necessarily reflect the policies or views of the organizations with which they are affiliated or the funding agencies. Funding organizations did not have a role in the study design, hiring or training of staff, intervention delivery, data collection, analysis, or data interpretation.

## Author contribution

ASW and SSS led FAARM's GAAP2 contribution. SG is the PI of the FAARM trial. ASW and JLW designed the data collection system with the support of SG. AK oversaw data collection activities. JLW processed and analyzed the data and prepared the tables and figures. JLW, ASW, and SSS wrote the first draft of the manuscript with the support of SG. All authors reviewed and revised the manuscript.

## Conflict of interest

The authors declare that they have no conflict of interest.

## Footnotes

2 An updated version of this questionnaire is available from IFPRI (International Food Policy Research Institute (IFPRI), 2022).
