# Supplementary material for: Impact of a homestead food production program on women's empowerment: Pro-WEAI results from the FAARM trial in Bangladesh
Source: World Dev. 2022 Oct;158:106001. doi: 10.1016/j.worlddev.2022.106001 (PMC9351289; doi:10.1016/j.worlddev.2022.106001)
Supplement: Supplementary data 1 [file mmc1.pdf]

# Impact of a homestead food production project on women's empowerment: Pro-WEAI results from the FAARM trial in Sylhet, Bangladesh

(Waid et al.)

## Online Supplemental Material

Page Item


| Page | Item |
|---|---|
| 3 | Supplemental Table S1: Comparison of the characteristics of the FAARM study population to women eligible for the pro-WEAI survey |
| 4 | Supplemental Table S2: Attainment of empowerment on each 3DE indicator, by sex of respondent and FAARM intervention group |
| 4 | Supplemental Table S3: Proportion who deem intimate partner violence acceptable in the listed conditions, by sex of respondent and FAARM intervention group |
| 4 | Supplemental Table S4: Proportion who state they use income as described in the vignette, by sex of respondent and FAARM intervention group |
| 5 | Supplemental Figure S1: Responses to the eight items on the new generalized self-efficacy scale, by sex of respondent and FAARM intervention group |
| 6 | Supplemental Figure S2: Responses to questions about inter-household trust and respect, by sex of respondent and FAARM intervention group |
| 7 | Supplemental Table S5: Credit availability, utilization, and decision-making, by sex of respondent and FAARM intervention group |
| 8 | Supplemental Table S6: Household and individual ownership of key assets, by sex of respondent and FAARM intervention group |
| 9 | Supplemental Figure S3: Density plot of the number of assets owned, by sex of respondent and FAARM intervention group |
| 10 | Supplemental Table S7: Participation in and decision-making on commodities production, by sex of respondent and FAARM intervention group |
| 11 | Supplemental Figure S4: Level of contribution to decision-making on consumption or income from productive on-farm activities, by sex of respondent and FAARM intervention group |
| 12 | Supplemental Figure S5: Level of contribution to decision-making on income from off-farm activities, by sex of respondent and FAARM intervention group |
| 12 | Supplemental Figure S6: Frequency of visiting the listed locations, by sex of respondent and FAARM intervention group |
| 13 | Supplemental Table S8: Average time in hours per day spent on the listed work and non-work activities, by sex of respondent and FAARM intervention group |
| 14 | Supplemental Table S9: Presence of and membership in community groups, by group type, sex of respondent, and FAARM intervention group |

15 Supplemental Figure S7 : Perceived influence of community groups to which the respondent is a member, by group type, sex of respondent, and FAARM intervention group

16 Supplemental Table S10: Impact of the FAARM intervention on Pro-WEAI indicators, by sex of respondent (without covariates)

17 Supplemental Figure S8: Density plot of the number of indicators in each domain for which empowerment was attained, by sex of respondent and FAARM intervention group

17 Supplemental Table S11: Mean number of indicators for which empowerment was attained, by pro-WEAI domain, sex of respondent, and FAARM intervention group

18 Supplemental Table S12: Full regression results for the impact of the FAARM intervention on pro-WEAI indicators

***Supplemental Table S1: Comparison of the characteristics of the FAARM study population to women eligible for the pro-WEAI survey***

| | FAARM study population | n | Eligible for pro-WEAI | n |
|---|---|---|---|---|
| Intervention group | 50% | 2619 | 48% | 2098 |
| Education | | | | |
| No education | 17% | 2616 | 18% | 2098 |
| Partial primary | 21% | 2616 | 22% | 2098 |
| Complete primary | 24% | 2616 | 23% | 2098 |
| Any secondary education | 38% | 2616 | 36% | 2098 |
| Household wealth tercile | | | | |
| Lower | 40% | 2607 | 47% | 2098 |
| Middle | 32% | 2607 | 32% | 2098 |
| Upper | 28% | 2607 | 21% | 2098 |
| Other household characteristics | | | | |
| Hindu | 31% | 2615 | 31% | 2098 |
| Nuclear family | 32% | 2607 | 40% | 2098 |
| Mean of continuous variables | | | | |
| Age | 18.1 | 2595 | 18.0 | 2093 |
| Household size | 7.1 | 2607 | 5.9 | 2098 |
| Years since marriage | 6.6 | 2595 | 6.7 | 2093 |

Note: All data are from the FAARM baseline survey (March - May 2015). Sample sizes vary slightly as we were unable to reach all respondents for all modules in the survey. The FAARM trial was undertaken in Habiganj District, Sylhet Division, Bangladesh.

***Supplemental Table S2: Attainment of empowerment on each 3DE indicator, by sex of respondent and FAARM intervention group***

| | Women | | | | Men | | | |
|---|---|---|---|---|---|---|---|---|
| | Control | | Intervention | | Control | | Intervention | |
| | % | *(95% CI)* | % | *(95% CI)* | % | *(95% CI)* | % | *(95% CI)* |
| *Intrinsic agency* | | | | | | | | |
| Intimate partner violence not acceptable | 67% | *(56-76)* | 85% | *(79-89)* | 89% | *(83-93)* | 89% | *(84-93)* |
| Autonomy in income | 56% | *(46-65)* | 63% | *(54-72)* | 91% | *(85-95)* | 96% | *(92-98)* |
| Self-efficacy | 32% | *(23-41)* | 55% | *(45-65)* | 35% | *(27-44)* | 52% | *(43-61)* |
| Respect among household members | 10% | *(6-16)* | 10% | *(6-17)* | 13% | *(8-21)* | 16% | *(11-23)* |
| *Instrumental agency* | | | | | | | | |
| Access to and decisions on financial services | 98% | *(95-99)* | 96% | *(92-98)* | 100% | *(97-100)* | 99% | *(96-100)* |
| Ownership of land and other assets | 75% | *(67-81)* | 85% | *(78-90)* | 99% | *(96-100)* | 99% | *(96-100)* |
| Input in productive decisions | 72% | *(63-79)* | 75% | *(66-83)* | 82% | *(73-88)* | 78% | *(69-85)* |
| Control over use of income | 78% | *(69-84)* | 84% | *(77-89)* | 68% | *(59-76)* | 56% | *(46-65)* |
| Visiting important locations | 48% | *(40-56)* | 54% | *(44-63)* | 60% | *(49-70)* | 70% | *(60-78)* |
| Work balance | 17% | *(12-23)* | 11% | *(8-16)* | 39% | *(32-47)* | 37% | *(31-43)* |
| *Collective agency* | | | | | | | | |
| Group membership | 30% | *(23-38)* | 80% | *(69-88)* | 53% | *(43-62)* | 58% | *(50-67)* |
| Membership in influential groups | 1% | *(0-3)* | 37% | *(25-49)* | 6% | *(3-11)* | 6% | *(4-11)* |
| Empowered in agency | 4% | *(2-8)* | 24% | *(17-32)* | 19% | *(13-26)* | 25% | *(19-32)* |

The FAARM trial was undertaken Habiganj District, Sylhet Division, Bangladesh. n=885

***Supplemental Table S3: Proportion who deem intimate partner violence acceptable in the listed conditions, by sex of respondent and FAARM intervention group***

| | Women | | | | Men | | | |
|---|---|---|---|---|---|---|---|---|
| | Control | | Intervention | | Control | | Intervention | |
| | % | *(95% CI)* | % | *(95% CI)* | % | *(95% CI)* | % | *(95% CI)* |
| Goes out without informing | 11% | *(7-17)* | 4% | *(2-8)* | 1% | *(0-4)* | 3% | *(2-6)* |
| Neglecting the children | 14% | *(9-20)* | 7% | *(4-11)* | 8% | *(5-13)* | 8% | *(5-13)* |
| Arguing | 23% | *(17-31)* | 10% | *(7-15)* | 4% | *(2-8)* | 5% | *(3-9)* |
| Refuses Sex | 4% | *(2-9)* | 1% | *(0-4)* | 0% | *(0-3)* | 0% | *(0-0)* |
| Burns food | 2% | *(1-5)* | 1% | *(0-4)* | 0% | *NA* | 0% | *NA* |

Confidence intervals cannot be calculated and are marked as not available (NA) when all observations in a given sex or intervention category were positive or negative. The FAARM trial was undertaken in Habiganj District, Sylhet Division, Bangladesh. n=885

***Supplemental Table S4: Proportion who state they use income as described in the vignette, by sex of respondent and FAARM intervention group***

| | Women | | | | Men | | | |
|---|---|---|---|---|---|---|---|---|
| | Control | | Intervention | | Control | | Intervention | |
| | % | *(95% CI)* | % | *(95% CI)* | % | *(95% CI)* | % | *(95% CI)* |
| No alternatives in income use | 61% | *(49-72)* | 54% | *(41-67)* | 79% | *(71-86)* | 79% | *(70-85)* |
| Uses income as told | 15% | *(9-23)* | 14% | *(9-22)* | 9% | *(5-15)* | 7% | *(4-14)* |
| Uses income as others expect | 1% | *(0-3)* | 1% | *(0-4)* | 17% | *(11-25)* | 17% | *(11-26)* |
| Uses income as personally wants | 56% | *(46-65)* | 63% | *(54-72)* | 93% | *(87-96)* | 97% | *(93-99)* |

Empowerment on autonomy on use of income was achieved when respondents stated that they used income as they personally wanted and did not use say that they both used income as told and as others expected (answering no to at least one of these two questions). The FAARM trial was undertaken in Habiganj District, Sylhet Division, Bangladesh. n=885

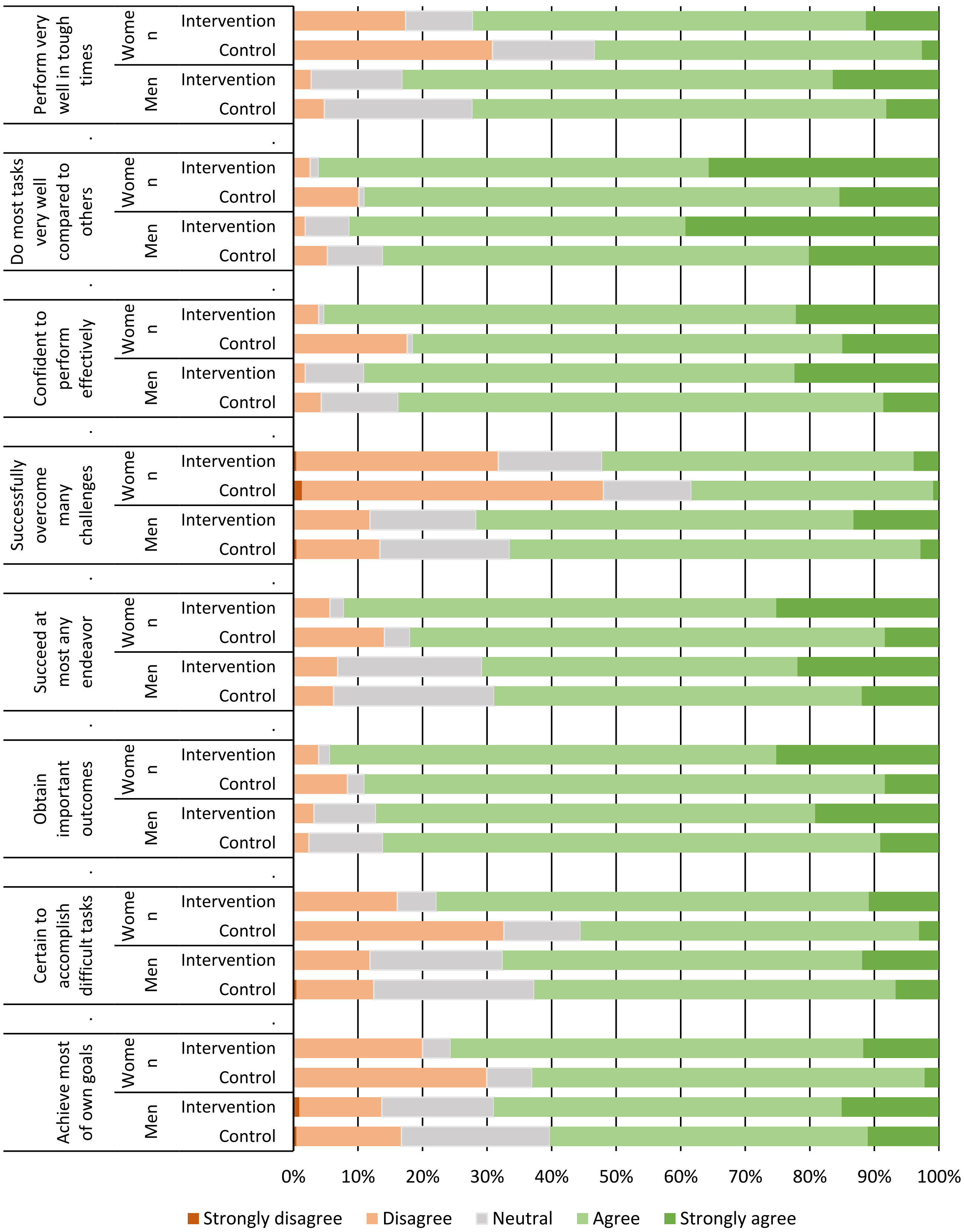


***Supplemental Figure S1: Responses to the eight items on the new generalized self-efficacy scale, by sex of respondent and FAARM intervention group***

Shades of orange indicator disagreement while shades of green indicate agreement. For all items more agreement equals higherself-efficacy. Empowerment was determined by an aggregate score of 32 out of 40. The FAARM trial was undertaken in Habiganj District, Sylhet Division, Bangladesh. n=885

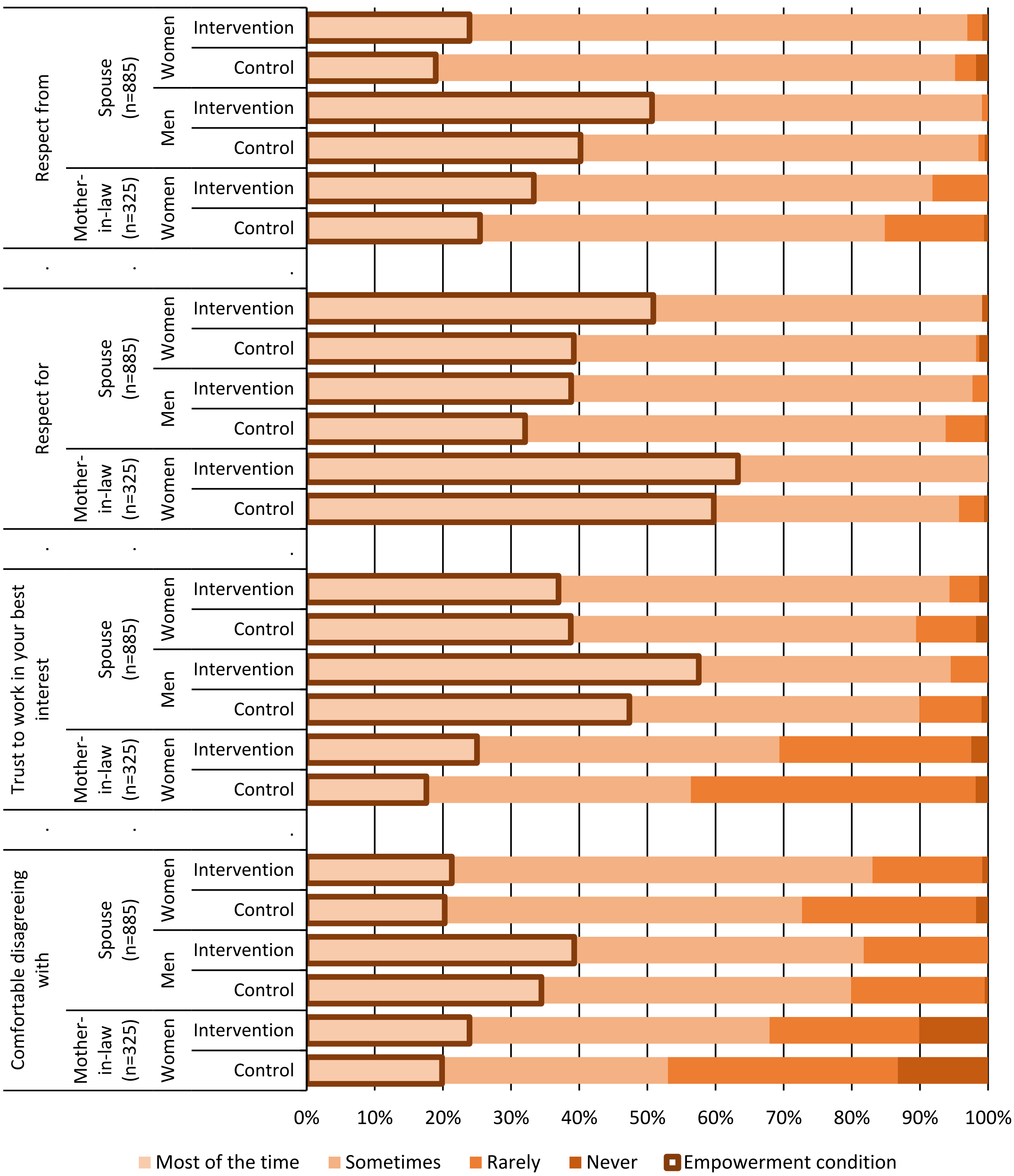


***Supplemental Figure S2: Responses to questions about inter-household trust and respect, by sex of respondent and FAARM intervention group***

Empowerment was attained when responses to all applicable questions were "most of the time," indicating that respect was both given and received and the respondent felt that the household member had their best interests in mind. Only women who lived with their mothers-in-law were asked the relevant questions. The FAARM trial was undertaken in Habiganj District, Sylhet Division, Bangladesh.

***Supplemental Table S5: Credit availability, utilization, and decision-making, by sex of respondent and FAARM intervention group***

| | Women | | | | Men | | | | |
|---|---|---|---|---|---|---|---|---|---|
| | Control | | Intervention | | Control | | Intervention | | n |
| | % | *(95% CI)* | % | *(95% CI)* | % | *(95% CI)* | % | *(95% CI)* | |
| NGO loan | | | | | | | | | 885 |
| Not available | 1% | *(0-4)* | 0% | *NA* | 1% | *(0-4)* | 0% | *NA* | |
| No loan in last year | 63% | *(54-70)* | 54% | *(47-61)* | 64% | *(56-71)* | 56% | *(49-63)* | |
| Cash loan taken | 36% | *(28-44)* | 46% | *(39-53)* | 35% | *(28-43)* | 44% | *(37-51)* | |
| In-kind loan taken | 0% | *(0-3)* | 0% | *NA* | 0% | *NA* | 0% | *NA* | |
| Respondent decided to borrow | 98% | *(90-99)* | 95% | *(89-98)* | 100% | *NA* | 98% | *(92-99)* | 355 |
| Respondent decided how to use | 80% | *(68-89)* | 71% | *(59-81)* | 100% | *NA* | 98% | *(92-99)* | 355 |
| Formal lender | | | | | | | | | 885 |
| Not available | 21% | *(15-28)* | 14% | *(9-21)* | 19% | *(13-26)* | 11% | *(6-18)* | |
| No loan in last year | 70% | *(62-78)* | 71% | *(63-78)* | 72% | *(63-79)* | 75% | *(66-83)* | |
| Cash loan taken | 9% | *(5-14)* | 15% | *(10-21)* | 9% | *(5-14)* | 14% | *(9-21)* | |
| In-kind loan taken | 0% | *NA* | 0% | *NA* | 1% | *(0-4)* | 0% | *NA* | |
| Respondent decided to borrow | 90% | *(69-97)* | 88% | *(69-96)* | 100% | *NA* | 94% | *(76-99)* | 105 |
| Respondent decided how to use | 65% | *(39-85)* | 62% | *(44-77)* | 100% | *NA* | 94% | *(76-99)* | 105 |
| Informal lender | | | | | | | | | 885 |
| Not available | 6% | *(3-12)* | 6% | *(3-11)* | 6% | *(3-15)* | 4% | *(2-9)* | |
| No loan in last year | 59% | *(51-67)* | 63% | *(55-69)* | 61% | *(53-68)* | 61% | *(54-68)* | |
| Cash loan taken | 35% | *(28-43)* | 31% | *(25-39)* | 33% | *(27-40)* | 35% | *(28-43)* | |
| In-kind loan taken | 0% | *NA* | 0% | *(0-3)* | 0% | *NA* | 0% | *NA* | |
| Respondent decided to borrow | 94% | *(80-98)* | 92% | *(81-97)* | 97% | *(82-100)* | 100% | *NA* | 297 |
| Respondent decided how to use | 67% | *(50-81)* | 68% | *(53-81)* | 97% | *(82-100)* | 100% | *NA* | 297 |
| Friends/relatives | | | | | | | | | 885 |
| Not available | 2% | *(1-5)* | 1% | *(0-3)* | 3% | *(1-6)* | 1% | *(0-4)* | |
| No loan in last year | 51% | *(43-58)* | 51% | *(43-59)* | 48% | *(39-58)* | 45% | *(37-53)* | |
| Cash loan taken | 48% | *(40-55)* | 48% | *(40-56)* | 49% | *(39-59)* | 54% | *(45-62)* | |
| In-kind loan taken | | | | | | | | | |
| Respondent decided to borrow | 92% | *(85-96)* | 91% | *(85-95)* | 99% | *(93-100)* | 100% | *NA* | 438 |
| Respondent decided how to use | 66% | *(55-75)* | 72% | *(60-81)* | 99% | *(93-100)* | 100% | *NA* | 438 |
| Formal group lending | | | | | | | | | 885 |
| Not available | 30% | *(20-42)* | 19% | *(12-29)* | 32% | *(21-45)* | 29% | *(19-41)* | |
| No loan in last year | 67% | *(56-77)* | 79% | *(69-86)* | 67% | *(54-78)* | 70% | *(59-80)* | |
| Cash loan taken | 3% | *(1-6)* | 2% | *(1-4)* | 1% | *(0-4)* | 1% | *(0-4)* | |
| In-kind loan taken | | | | | | | | | |
| Respondent decided to borrow | 67% | *(28-91)* | 100% | *NA* | 100% | *NA* | 100% | *NA* | 15 |
| Respondent decided how to use | 67% | *(28-91)* | 50% | *(9-91)* | 100% | *NA* | 100% | *NA* | 15 |
| Informal group lending | | | | | | | | | 885 |
| Not available | 45% | *(33-58)* | 38% | *(26-51)* | 50% | *(38-62)* | 41% | *(29-53)* | |
| No loan in last year | 55% | *(42-67)* | 59% | *(46-70)* | 50% | *(38-62)* | 55% | *(44-66)* | |
| Cash loan taken | 0% | *(0-3)* | 3% | *(1-9)* | 0% | *NA* | 4% | *(2-10)* | |
| In-kind loan taken | | | | | | | | | |
| Respondent decided to borrow | 100% | *NA* | 100% | *NA* | | *NA* | 100% | *NA* | 18 |
| Respondent decided how to use | 100% | *NA* | 100% | *NA* | | *NA* | 100% | *NA* | 18 |

Confidence intervals cannot be calculated and are marked as not available (NA) when all observations in a given sex or intervention category were positive or negative. The FAARM trial was undertaken in Habiganj District, Sylhet Division, Bangladesh.

***Supplemental Table S6: Household and individual ownership of key assets, by sex of respondent and FAARM intervention group***

| | Women | | | | Men | | | |
|---|---|---|---|---|---|---|---|---|
| | Control | | Intervention | | Control | | Intervention | |
| | % | *(95% CI)* | % | *(95% CI)* | % | *(95% CI)* | % | *(95% CI)* |
| Agricultural land | | | | | | | | |
| Household doesn't own | 21% | *(16-28)* | 14% | *(9-21)* | 18% | *(13-25)* | 16% | *(11-23)* |
| Respondent sole ownership | 0% | *(0-3)* | 1% | *(0-3)* | 32% | *(24-40)* | 33% | *(25-43)* |
| Respondent joint ownership | 2% | *(1-5)* | 1% | *(0-4)* | 4% | *(2-8)* | 5% | *(2-9)* |
| Respondent sole and joint ownership | 0% | *(0-3)* | 1% | *(0-4)* | 3% | *(1-6)* | 2% | *(1-5)* |
| Respondent does not own | 76% | *(69-82)* | 82% | *(75-87)* | 44% | *(35-53)* | 43% | *(35-52)* |
| Large livestock | | | | | | | | |
| Household doesn't own | 59% | *(52-65)* | 60% | *(52-68)* | 56% | *(49-62)* | 57% | *(49-64)* |
| Respondent sole ownership | 2% | *(1-5)* | 3% | *(2-6)* | 24% | *(18-31)* | 21% | *(16-28)* |
| Respondent joint ownership | 13% | *(8-19)* | 7% | *(5-11)* | 12% | *(8-18)* | 13% | *(9-19)* |
| Respondent sole and joint ownership | 2% | *(1-5)* | 0% | *(0-3)* | 0% | *(0-3)* | 0% | *(0-3)* |
| Respondent does not own | 25% | *(20-31)* | 29% | *(22-36)* | 7% | *(4-11)* | 8% | *(5-13)* |
| Small livestock | | | | | | | | |
| Household doesn't own | 83% | *(77-88)* | 81% | *(75-85)* | 83% | *(76-89)* | 81% | *(75-86)* |
| Respondent sole ownership | 3% | *(2-6)* | 6% | *(3-9)* | 5% | *(2-9)* | 8% | *(5-12)* |
| Respondent joint ownership | 5% | *(3-9)* | 6% | *(3-11)* | 3% | *(1-8)* | 3% | *(2-6)* |
| Respondent sole and joint ownership | 1% | *(0-6)* | 1% | *(0-5)* | 0% | *NA* | 0% | *(0-3)* |
| Respondent does not own | 8% | *(5-12)* | 7% | *(4-10)* | 9% | *(5-14)* | 8% | *(5-13)* |
| Poultry | | | | | | | | |
| Household doesn't own | 35% | *(28-43)* | 22% | *(16-28)* | 35% | *(28-44)* | 22% | *(16-30)* |
| Respondent sole ownership | 36% | *(29-43)* | 47% | *(39-55)* | 7% | *(4-12)* | 10% | *(5-18)* |
| Respondent joint ownership | 17% | *(11-25)* | 19% | *(13-27)* | 15% | *(11-21)* | 16% | *(11-22)* |
| Respondent sole and joint ownership | 7% | *(4-10)* | 7% | *(4-14)* | 0% | *NA* | 0% | *NA* |
| Respondent does not own | 6% | *(3-11)* | 5% | *(3-9)* | 43% | *(34-52)* | 52% | *(44-60)* |
| Fish pond | | | | | | | | |
| Household doesn't own | 23% | *(18-30)* | 18% | *(13-25)* | 11% | *(8-17)* | 11% | *(7-16)* |
| Respondent sole ownership | 1% | *(0-3)* | 0% | *(0-3)* | 70% | *(62-77)* | 65% | *(57-72)* |
| Respondent joint ownership | 3% | *(2-6)* | 2% | *(1-4)* | 15% | *(10-22)* | 20% | *(14-26)* |
| Respondent sole and joint ownership | 0% | *(0-3)* | 0% | *(0-3)* | 0% | *NA* | 0% | *NA* |
| Respondent does not own | 72% | *(66-78)* | 79% | *(72-85)* | 3% | *(1-7)* | 5% | *(2-12)* |
| Non-mechanized farm equipment | | | | | | | | |
| Household doesn't own | 6% | *(3-10)* | 3% | *(1-6)* | 5% | *(3-9)* | 7% | *(3-14)* |
| Respondent sole ownership | 4% | *(2-8)* | 6% | *(4-10)* | 77% | *(69-83)* | 56% | *(46-66)* |
| Respondent joint ownership | 6% | *(3-10)* | 4% | *(2-8)* | 16% | *(10-23)* | 24% | *(17-32)* |
| Respondent sole and joint ownership | 3% | *(1-6)* | 33% | *(23-46)* | 0% | *NA* | 10% | *(5-17)* |
| Respondent does not own | 82% | *(76-86)* | 53% | *(42-64)* | 2% | *(1-5)* | 4% | *(2-8)* |
| Mechanized farm equipment | | | | | | | | |
| Household doesn't own | 86% | *(81-90)* | 87% | *(80-91)* | 86% | *(81-90)* | 88% | *(82-92)* |
| Respondent sole ownership | 0% | *(0-3)* | 0% | *(0-3)* | 8% | *(5-12)* | 8% | *(5-13)* |
| Respondent joint ownership | 0% | *(0-3)* | 0% | *(0-3)* | 4% | *(2-8)* | 3% | *(2-6)* |
| Respondent sole and joint ownership | 0% | *NA* | 0% | *(0-3)* | 0% | *NA* | 0% | *NA* |
| Respondent does not own | 13% | *(9-18)* | 12% | *(8-18)* | 2% | *(1-6)* | 1% | *(0-4)* |
| Non-farm equipment | | | | | | | | |
| Household doesn't own | 73% | *(65-80)* | 73% | *(65-80)* | 78% | *(70-84)* | 73% | *(64-80)* |
| Respondent sole ownership | 8% | *(5-13)* | 7% | *(4-12)* | 10% | *(6-15)* | 15% | *(9-23)* |
| Respondent joint ownership | 0% | *(0-3)* | 0% | *(0-3)* | 5% | *(3-10)* | 7% | *(4-12)* |
| Respondent sole and joint ownership | 0% | *NA* | 0% | *(0-3)* | 0% | *NA* | 0% | *NA* |
| Respondent does not own | 18% | *(12-26)* | 18% | *(12-27)* | 7% | *(4-12)* | 5% | *(3-10)* |
| Building | | | | | | | | |
| Household doesn't own | 1% | *(0-3)* | 0% | *(0-3)* | 1% | *(0-4)* | 1% | *(0-4)* |
| Respondent sole ownership | 2% | *(1-5)* | 1% | *(0-4)* | 64% | *(56-72)* | 63% | *(55-70)* |
| Respondent joint ownership | 3% | *(2-6)* | 1% | *(0-3)* | 18% | *(11-28)* | 19% | *(13-26)* |
| Respondent sole and joint ownership | 0% | *NA* | 0% | *NA* | 0% | *NA* | 0% | *NA* |
| Respondent does not own | 94% | *(90-97)* | 97% | *(94-99)* | 17% | *(12-23)* | 17% | *(12-24)* |

***Supplemental Table S6: Household and individual ownership of key assets, by sex of respondent and FAARM intervention group (continued)***

| | Women | | | | Men | | | |
|---|---|---|---|---|---|---|---|---|
| | Control | | Intervention | | Control | | Intervention | |
| | % | *(95% CI)* | % | *(95% CI)* | % | *(95% CI)* | % | *(95% CI)* |
| Large consumer durables | | | | | | | | |
| Household doesn't own | 4% | *(2-9)* | 2% | *(1-6)* | 1% | *(0-4)* | 2% | *(1-6)* |
| Respondent sole ownership | 11% | *(7-17)* | 13% | *(9-20)* | 22% | *(16-29)* | 21% | *(16-28)* |
| Respondent joint ownership | 41% | *(30-53)* | 31% | *(21-42)* | 73% | *(65-79)* | 70% | *(62-76)* |
| Respondent sole and joint ownership | 21% | *(13-32)* | 30% | *(21-41)* | 0% | *NA* | 3% | *(1-7)* |
| Respondent does not own | 23% | *(16-32)* | 24% | *(16-34)* | 4% | *(2-8)* | 4% | *(2-7)* |
| Small consumer durables | | | | | | | | |
| Household doesn't own | 0% | *NA* | 0% | *NA* | 0% | *(0-3)* | 0% | *(0-3)* |
| Respondent sole ownership | 32% | *(23-42)* | 34% | *(25-46)* | 9% | *(5-15)* | 9% | *(5-16)* |
| Respondent joint ownership | 39% | *(28-51)* | 38% | *(27-50)* | 77% | *(70-83)* | 72% | *(62-80)* |
| Respondent sole and joint ownership | 22% | *(16-31)* | 22% | *(15-30)* | 0% | *(0-3)* | 0% | *NA* |
| Respondent does not own | 7% | *(4-13)* | 6% | *(3-12)* | 13% | *(8-21)* | 19% | *(11-29)* |
| Mobile phone | | | | | | | | |
| Household doesn't own | 3% | *(1-6)* | 4% | *(2-7)* | 2% | *(1-6)* | 3% | *(1-6)* |
| Respondent sole ownership | 18% | *(13-25)* | 16% | *(11-23)* | 90% | *(84-94)* | 90% | *(85-94)* |
| Respondent joint ownership | 17% | *(11-24)* | 14% | *(9-22)* | 4% | *(2-8)* | 4% | *(2-10)* |
| Respondent sole and joint ownership | 22% | *(15-31)* | 22% | *(15-31)* | 1% | *(0-4)* | 0% | *NA* |
| Respondent does not own | 41% | *(32-50)* | 44% | *(36-52)* | 2% | *(1-6)* | 3% | *(1-6)* |
| Non-Agricultural land | | | | | | | | |
| Household doesn't own | 4% | *(1-10)* | 2% | *(1-4)* | 4% | *(2-8)* | 2% | *(1-5)* |
| Respondent sole ownership | 1% | *(0-3)* | 0% | *(0-3)* | 56% | *(48-64)* | 59% | *(51-66)* |
| Respondent joint ownership | 2% | *(1-5)* | 1% | *(0-3)* | 14% | *(8-23)* | 14% | *(10-19)* |
| Respondent sole and joint ownership | 0% | *NA* | 0% | *(0-3)* | 0% | *NA* | 0% | *NA* |
| Respondent does not own | 93% | *(88-97)* | 97% | *(93-98)* | 26% | *(20-33)* | 25% | *(19-32)* |
| Vehicle(s) | | | | | | | | |
| Household doesn't own | 84% | *(77-89)* | 77% | *(71-83)* | 84% | *(78-89)* | 79% | *(73-85)* |
| Respondent sole ownership | 0% | *NA* | 0% | *NA* | 11% | *(7-17)* | 16% | *(12-20)* |
| Respondent joint ownership | 0% | *(0-3)* | 0% | *(0-3)* | 1% | *(0-4)* | 1% | *(0-4)* |
| Respondent sole and joint ownership | 0% | *NA* | 0% | *NA* | 0% | *(0-3)* | 0% | *NA* |
| Respondent does not own | 16% | *(11-22)* | 22% | *(17-29)* | 3% | *(1-6)* | 4% | *(2-8)* |

Confidence intervals cannot be calculated and are marked as not available (NA) when all observations in a given sex or intervention category were positive or negative. The FAARM trial was undertaken in Habiganj District, Sylhet Division, Bangladesh. n=885

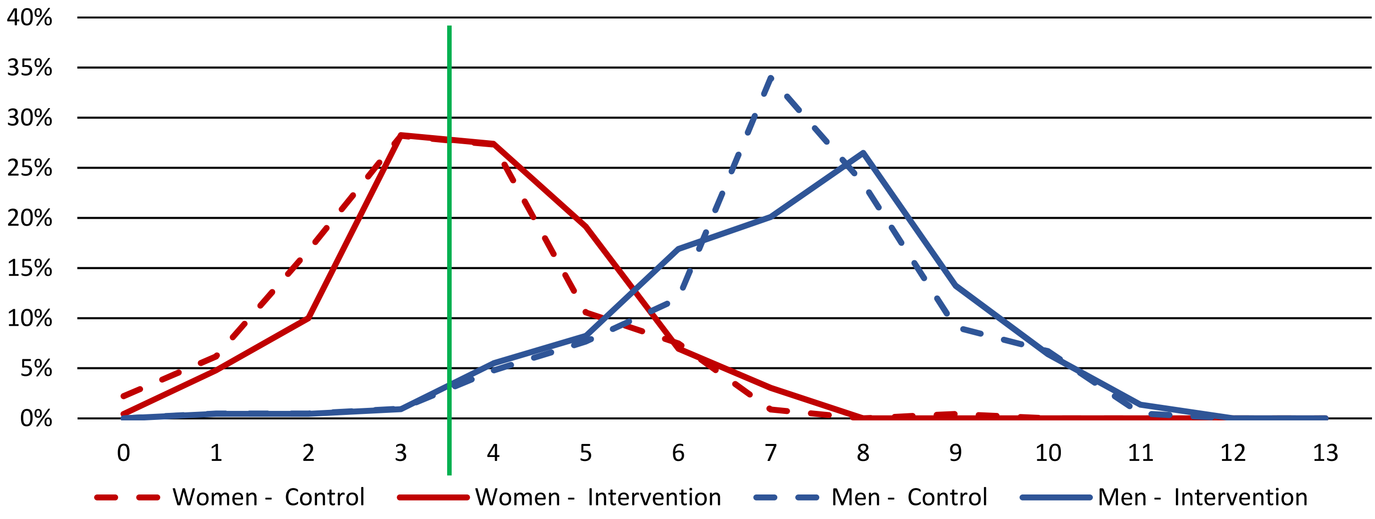


***Supplemental Figure S3: Density plot of the number of assets owned, by sex of respondent and FAARM intervention group***

The proportion of respondents, by sex and intervention, who own the number of assets given on the x-axis. The number excludes land. Blue is for men and red for women, while the dashed lines are for control group and the solid lines for the intervention group. A respondent was considered empowered when they at least partially owned three or more assets, as indicated by the vertical green line, or land (see Supplemental Table 6). The FAARM trial was undertaken in Habiganj District, Sylhet Division, Bangladesh. n=885

***Supplemental Table S7: Participation in and decision-making on commodities production by sex of respondent and FAARM intervention group***

| | Women | | | | Men | | | | |
|---|---|---|---|---|---|---|---|---|---|
| | Control | | Intervention | | Control | | Intervention | | n |
| | % | *(95% CI)* | % | *(95% CI)* | % | *(95% CI)* | % | *(95% CI)* | |
| Staple grain | | | | | | | | | |
| Participates in | 75% | *(68-81)* | 81% | *(75-87)* | 79% | *(72-85)* | 84% | *(78-89)* | 885 |
| Of participant: Solely makes decision | 1% | *(0-4)* | 1% | *(0-4)* | 1% | *(0-5)* | 1% | *(0-4)* | 707 |
| Of participant, not sole decision maker: | | | | | | | | | |
| Greater than little input into decision | 90% | *(84-94)* | 92% | *(85-96)* | 99% | *(96-100)* | 100% | *(0-0)* | 702 |
| Possible input more than small | 7% | *(4-13)* | 17% | *(11-24)* | 98% | *(94-99)* | 97% | *(92-99)* | 702 |
| Horticulture | | | | | | | | | |
| Participates in | 81% | *(74-86)* | 96% | *(93-98)* | 80% | *(74-85)* | 96% | *(92-98)* | 885 |
| Of participant: Solely makes decision | 6% | *(3-10)* | 7% | *(4-13)* | 1% | *(0-4)* | 0% | *NA* | 782 |
| Of participant, not sole decision maker: | | | | | | | | | |
| Greater than little input into decision | 98% | *(94-99)* | 98% | *(95-99)* | 89% | *(81-93)* | 79% | *(68-86)* | 754 |
| Possible input more than small | 72% | *(62-80)* | 89% | *(83-93)* | 77% | *(67-84)* | 79% | *(70-86)* | 754 |
| Large livestock | | | | | | | | | |
| Participates in | 45% | *(39-52)* | 47% | *(39-55)* | 48% | *(41-55)* | 49% | *(42-57)* | 885 |
| Of participant: Solely makes decision | 1% | *(0-7)* | 0% | *NA* | 0% | *NA* | 0% | *NA* | 420 |
| Of participant, not sole decision maker: | | | | | | | | | |
| Greater than little input into decision | 92% | *(84-96)* | 93% | *(84-97)* | 99% | *(93-100)* | 100% | *NA* | 419 |
| Possible input more than small | 42% | *(30-55)* | 49% | *(38-59)* | 89% | *(79-94)* | 96% | *(89-99)* | 419 |
| Small Livestock | | | | | | | | | |
| Participates in | 19% | *(13-25)* | 21% | *(16-27)* | 18% | *(13-25)* | 22% | *(17-28)* | 885 |
| Of participant: Solely makes decision | 2% | *(0-14)* | 4% | *(1-15)* | 0% | *NA* | 0% | *NA* | 177 |
| Of participant, not sole decision maker: | | | | | | | | | |
| Greater than little input into decision | 90% | *(76-96)* | 98% | *(85-100)* | 97% | *(84-100)* | 94% | *(82-98)* | 174 |
| Possible input more than small | 59% | *(42-73)* | 79% | *(66-88)* | 87% | *(69-95)* | 88% | *(75-94)* | 174 |
| Poultry | | | | | | | | | |
| Participates in | 71% | *(64-78)* | 86% | *(78-91)* | 70% | *(61-77)* | 87% | *(80-92)* | 885 |
| Of participant: Solely makes decision | 15% | *(10-23)* | 13% | *(7-22)* | 1% | *(0-5)* | 1% | *(0-4)* | 696 |
| Of participant, not sole decision maker: | | | | | | | | | |
| Greater than little input into decision | 99% | *(94-100)* | 99% | *(92-100)* | 70% | *(58-79)* | 67% | *(55-77)* | 644 |
| Possible input more than small | 85% | *(78-90)* | 96% | *(92-98)* | 48% | *(37-60)* | 52% | *(42-61)* | 644 |
| Fishpond | | | | | | | | | |
| Participates in | 67% | *(60-73)* | 70% | *(63-76)* | 84% | *(78-89)* | 89% | *(83-92)* | 885 |
| Of participant: Solely makes decision | 3% | *(1-7)* | 1% | *(0-4)* | 11% | *(7-18)* | 3% | *(1-7)* | 683 |
| Of participant, not sole decision maker: | | | | | | | | | |
| Greater than little input into decision | 66% | *(54-76)* | 70% | *(58-80)* | 100% | *NA* | 100% | *NA* | 651 |
| Possible input more than small | 7% | *(3-13)* | 8% | *(5-12)* | 98% | *(92-100)* | 97% | *(92-99)* | 651 |

Confidence intervals cannot be calculated and are marked as not available (NA) when all observations in a given sex or intervention category were positive or negative. The FAARM trial was undertaken in Habiganj District, Sylhet Division, Bangladesh.

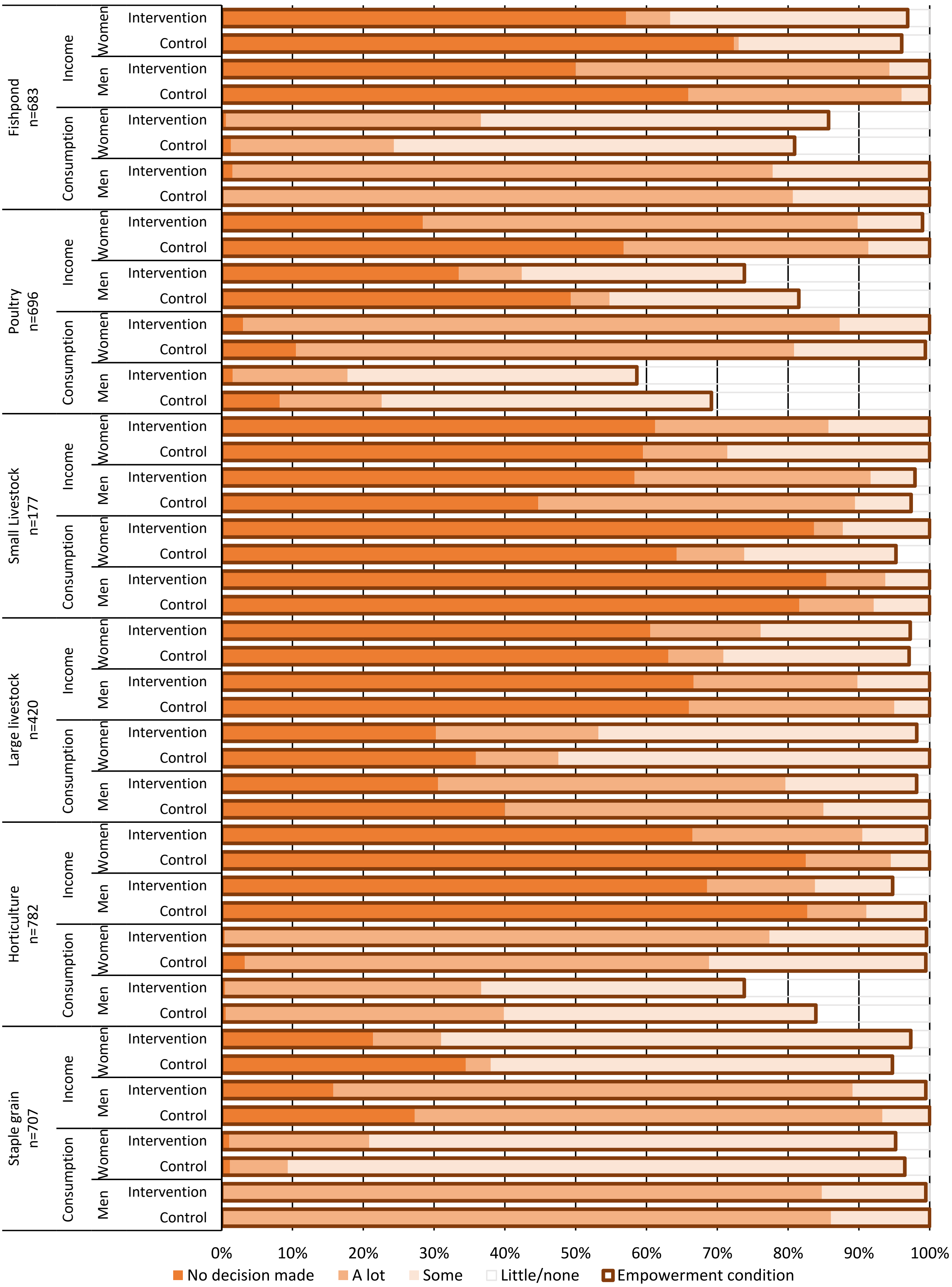


***Supplemental Figure S4: Level of contribution to decision-making on consumption or income from productive on-farm activities in which the respondent participated, by sex of respondent and FAARM intervention group***

Empowerment on control over use of income was defined as respondents with at least some decision-making power on all activities in which they participated both on-farm (this figure) and off-farm (Supplemental Figure 5).The FAARM trial was undertaken in Habiganj District, Sylhet Division, Bangladesh.

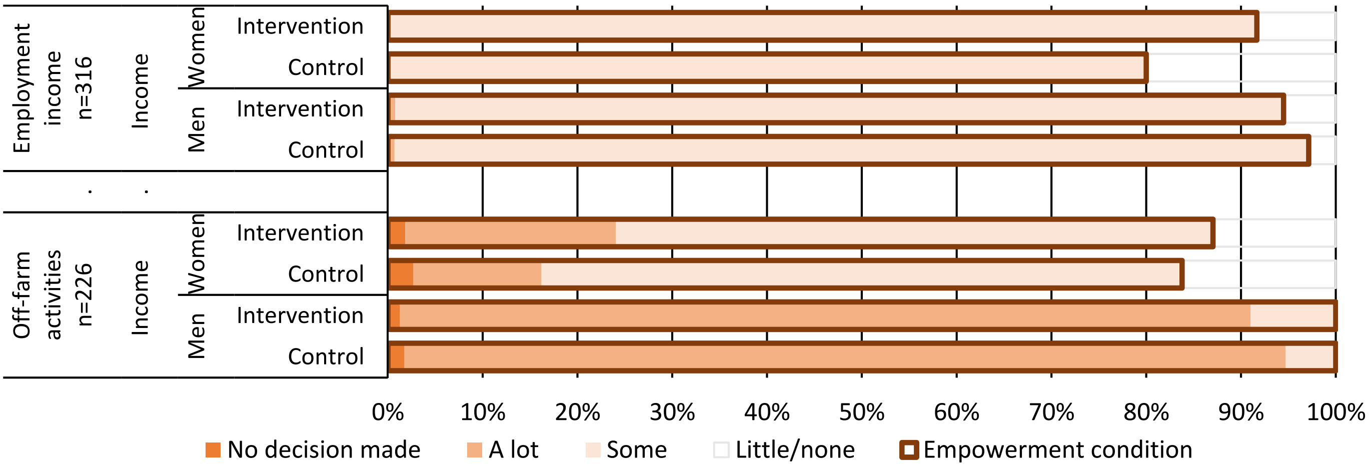


***Supplemental Figure S5: Level of contribution to decision-making on income from off-farm activities in which the respondent participated, by sex of respondent and FAARM intervention group***

Empowerment on control over use of income was achieved when respondents had at least some decision-making power on all activities in which they participated both on-farm (Supplemental Figure 4) and off-farm (this figure). The FAARM trial was undertaken Habiganj District, Sylhet Division, Bangladesh.

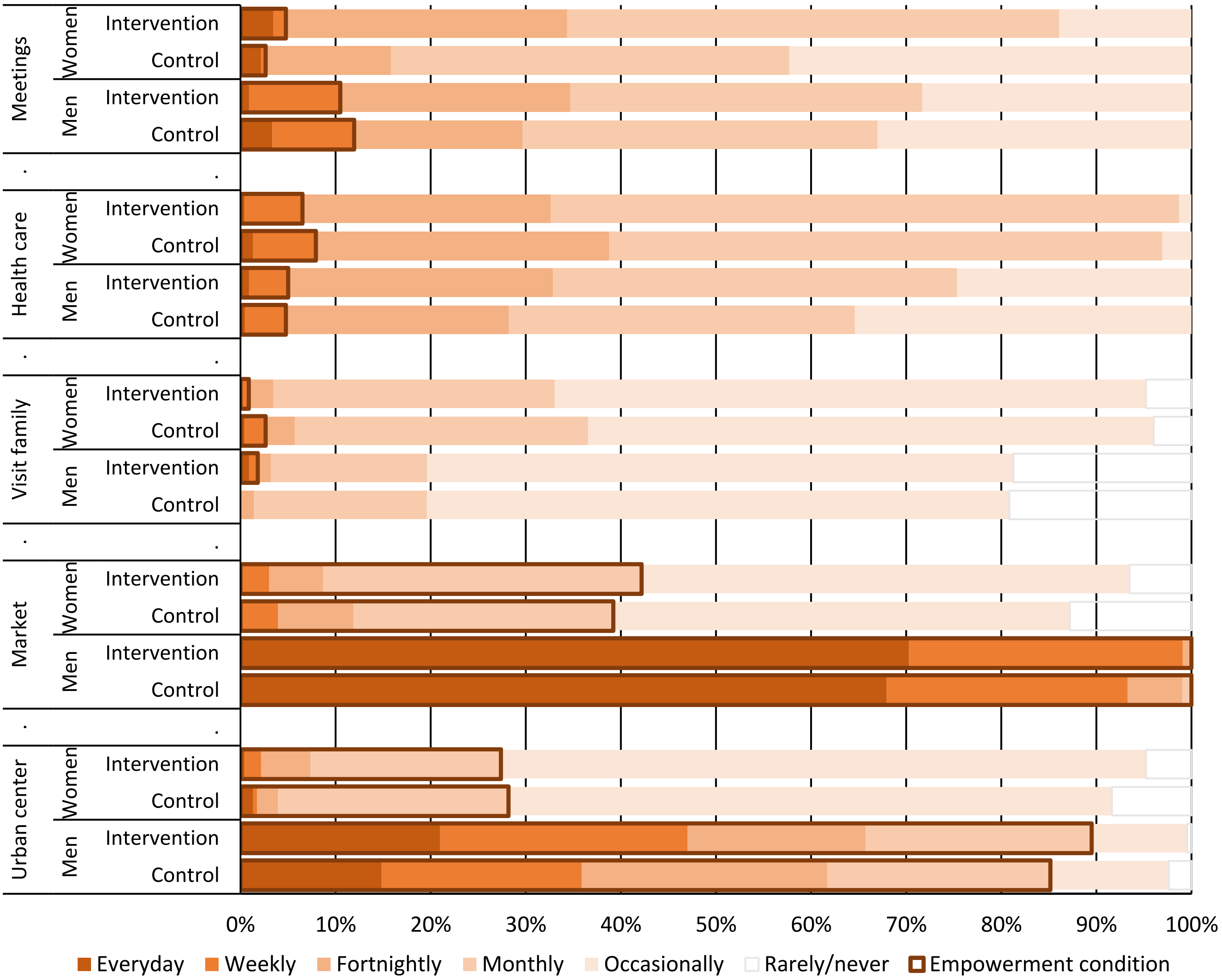


***Supplemental Figure S6: Frequency of visiting the listed locations, by sex of respondent and FAARM intervention group***

Empowerment in mobility was achieved when the respondent visited at least two of the three locations weekly (meetings, health care facilities, and family) and at least one of two locations monthly (market and urban center). The FAARM trial was undertaken in Habiganj District, Sylhet Division, Bangladesh. n=885

***Supplemental Table S8: Average time in hours per day spent on the listed work and non-work activities, by sex of respondent and FAARM intervention group***

| | Women | | | | Men | | | |
|---|---|---|---|---|---|---|---|---|
| | Control | | Intervention | | Control | | Intervention | |
| | mean | *(95% CI)* | mean | *(95% CI)* | mean | *(95% CI)* | mean | *(95% CI)* |
| ***Non-Work*** | ***14.7*** | ***(14.1-15.2)*** | ***14.7*** | ***(14.0-15.3)*** | ***14.6*** | ***(14.1-15.1)*** | ***14.2*** | ***(13.8-14.7)*** |
| Resting | 9.7 | *(9.3-10.1)* | 9.7 | *(9.4-10.1)* | 9.6 | *(9.2-9.9)* | 9.5 | *(9.2-9.8)* |
| Eating | 1.8 | *(1.6-2.0)* | 1.7 | *(1.5-1.9)* | 1.6 | *(1.5-1.7)* | 1.6 | *(1.5-1.7)* |
| Personal care | 1.1 | *(1.0-1.2)* | 1.1 | *(1.0-1.1)* | 1.0 | *(0.9-1.1)* | 1.0 | *(0.9-1.0)* |
| Study | 0.6 | *(0.3-0.9)* | 0.7 | *(0.4-1.1)* | 0.0 | *(0.0-0.0)* | 0.0 | *(0.0-0.0)* |
| Other travel | 0.3 | *(0.1-0.4)* | 0.2 | *(0.1-0.4)* | 1.0 | *(0.7-1.3)* | 0.8 | *(0.6-1.1)* |
| Exercise | 0.0 | *NA* | 0.0 | *(0.0-0.0)* | 0.0 | *(0.0-0.0)* | 0.0 | *NA* |
| Social/hobbies | 0.8 | *(0.5-1.0)* | 0.9 | *(0.6-1.1)* | 1.1 | *(0.8-1.4)* | 1.0 | *(0.8-1.3)* |
| Religion | 0.5 | *(0.3-0.6)* | 0.3 | *(0.2-0.5)* | 0.3 | *(0.2-0.5)* | 0.3 | *(0.2-0.4)* |
| Other | 0.0 | *(0.0-0.0)* | 0.0 | *(0.0-0.0)* | 0.0 | *(0.0-0.1)* | 0.0 | *(0.0-0.0)* |
| ***Work*** | ***9.3*** | ***(8.8-9.9)*** | ***9.3*** | ***(8.7-10.0)*** | ***9.4*** | ***(8.9-9.9)*** | ***9.8*** | ***(9.3-10.2)*** |
| Job | 0.3 | *(0.0-0.5)* | 0.2 | *(0.0-0.5)* | 3.6 | *(2.9-4.3)* | 3.5 | *(2.9-4.1)* |
| Own business | 0.1 | *(0.0-0.2)* | 0.0 | *(0.0-0.1)* | 1.4 | *(0.9-1.8)* | 1.8 | *(1.3-2.3)* |
| Staple grain | 1.6 | *(1.1-2.0)* | 1.3 | *(0.8-1.7)* | 2.4 | *(1.7-3.0)* | 2.6 | *(2.0-3.2)* |
| Horticulture | 0.1 | *(0.0-0.2)* | 0.2 | *(0.0-0.4)* | 0.1 | *(0.0-0.2)* | 0.0 | *(0.0-0.1)* |
| Large livestock | 0.2 | *(0.2-0.3)* | 0.1 | *(0.1-0.2)* | 0.4 | *(0.3-0.6)* | 0.4 | *(0.2-0.5)* |
| Small livestock | 0.0 | *(0.0-0.1)* | 0.0 | *(0.0-0.1)* | 0.0 | *(0.0-0.0)* | 0.0 | *(0.0-0.0)* |
| Poultry | 0.1 | *(0.0-0.1)* | 0.1 | *(0.1-0.2)* | 0.0 | *NA* | 0.0 | *(0.0-0.0)* |
| Aquaculture | 0.0 | *(0.0-0.0)* | 0.0 | *(0.0-0.0)* | 0.1 | *(0.0-0.2)* | 0.0 | *(0.0-0.1)* |
| Commuting | 0.0 | *(0.0-0.0)* | 0.0 | *(0.0-0.0)* | 0.0 | *(0.0-0.0)* | 0.0 | *(0.0-0.0)* |
| Marketing | 0.0 | *(0.0-0.1)* | 0.0 | *(0.0-0.1)* | 0.6 | *(0.5-0.8)* | 0.7 | *(0.5-0.8)* |
| Textile care | 0.2 | *(0.1-0.4)* | 0.1 | *(0.0-0.2)* | 0.0 | *NA* | 0.0 | *NA* |
| Cooking | 2.9 | *(2.8-3.1)* | 2.9 | *(2.8-3.1)* | 0.0 | *NA* | 0.0 | *NA* |
| Domestic work | 2.2 | *(1.8-2.6)* | 2.4 | *(1.9-2.8)* | 0.3 | *(0.1-0.4)* | 0.2 | *(0.1-0.3)* |
| Child care (primary activity) | 1.4 | *(1.1-1.6)* | 1.7 | *(1.5-1.9)* | 0.4 | *(0.3-0.5)* | 0.4 | *(0.3-0.6)* |
| Adult member care | 0.2 | *(0.1-0.3)* | 0.1 | *(0.0-0.2)* | 0.1 | *(0.0-0.2)* | 0.0 | *(0.0-0.0)* |
| ***Child care as a secondary activity*** | ***10.5*** | ***(9.3-11.8)*** | ***10.9*** | ***(9.7-12.0)*** | ***2.2*** | ***(1.7-2.6)*** | ***2.0*** | ***(1.6-2.4)*** |

Confidence intervals cannot be calculated and are marked as not available (NA) when all observations in a given sex or intervention category were zero. Empowerment in work balance was achieved when work was less than 10.5 hours with "childcare as a secondary activity" being counted at half of the time spent. The FAARM trial was undertaken in Habiganj District, Sylhet Division, Bangladesh. n=885

***Supplemental Table S9: Presence of and membership in community groups, by group type, sex of respondent, and FAARM intervention group***

| | Women | | | | Men | | | | |
|---|---|---|---|---|---|---|---|---|---|
| | Control | | Intervention | | Control | | Intervention | | n |
| | % | *(95% CI)* | % | *(95% CI)* | % | *(95% CI)* | % | *(95% CI)* | |
| Producers' group | | | | | | | | | |
| Present in community | 85% | *(73-92)* | 95% | *(88-98)* | 87% | *(74-94)* | 97% | *(91-99)* | 885 |
| Member of group | 4% | *(1-11)* | 78% | *(65-87)* | 27% | *(19-37)* | 23% | *(16-32)* | 804 |
| Water users' group | | | | | | | | | |
| Present in community | 48% | *(34-61)* | 44% | *(31-58)* | 49% | *(36-63)* | 47% | *(34-60)* | 885 |
| Member of group | 0% | *NA* | 1% | *(0-7)* | 19% | *(11-31)* | 18% | *(11-28)* | 414 |
| Forest users' group | | | | | | | | | |
| Present in community | 2% | *(0-14)* | 6% | *(2-17)* | 2% | *(0-16)* | 6% | *(2-17)* | 885 |
| Member of group | 0% | *NA* | 0% | *NA* | 20% | *(20-20)* | 0% | *NA* | 37 |
| Microfinance group | | | | | | | | | |
| Present in community | 92% | *(83-96)* | 93% | *(86-97)* | 100% | *(97-100)* | 99% | *(91-100)* | 885 |
| Member of group | 31% | *(24-39)* | 41% | *(33-50)* | 1% | *(0-4)* | 5% | *(3-9)* | 846 |
| Insurance group | | | | | | | | | |
| Present in community | 12% | *(6-25)* | 13% | *(6-25)* | 8% | *(3-19)* | 11% | *(5-22)* | 885 |
| Member of group | 0% | *NA* | 3% | *(0-26)* | 29% | *(12-55)* | 35% | *(18-56)* | 98 |
| Trade group | | | | | | | | | |
| Present in community | 22% | *(13-35)* | 17% | *(9-31)* | 24% | *(14-38)* | 19% | *(10-32)* | 885 |
| Member of group | 0% | *NA* | 0% | *NA* | 16% | *(9-28)* | 15% | *(7-27)* | 182 |
| Civic group | | | | | | | | | |
| Present in community | 43% | *(30-56)* | 43% | *(30-56)* | 49% | *(35-62)* | 44% | *(31-58)* | 885 |
| Member of group | 1% | *(0-7)* | 0% | *NA* | 24% | *(14-37)* | 21% | *(12-33)* | 394 |
| Religious group | | | | | | | | | |
| Present in community | 97% | *(88-99)* | 90% | *(80-96)* | 99% | *(96-100)* | 100% | *(97-100)* | 885 |
| Member of group | 0% | *NA* | 2% | *(1-8)* | 39% | *(30-48)* | 39% | *(32-46)* | 853 |

Confidence intervals cannot be calculated and are marked as not available (NA) when all observations in a given sex or intervention category were positive or negative. The FAARM trial was undertaken in Habiganj District, Sylhet Division, Bangladesh.

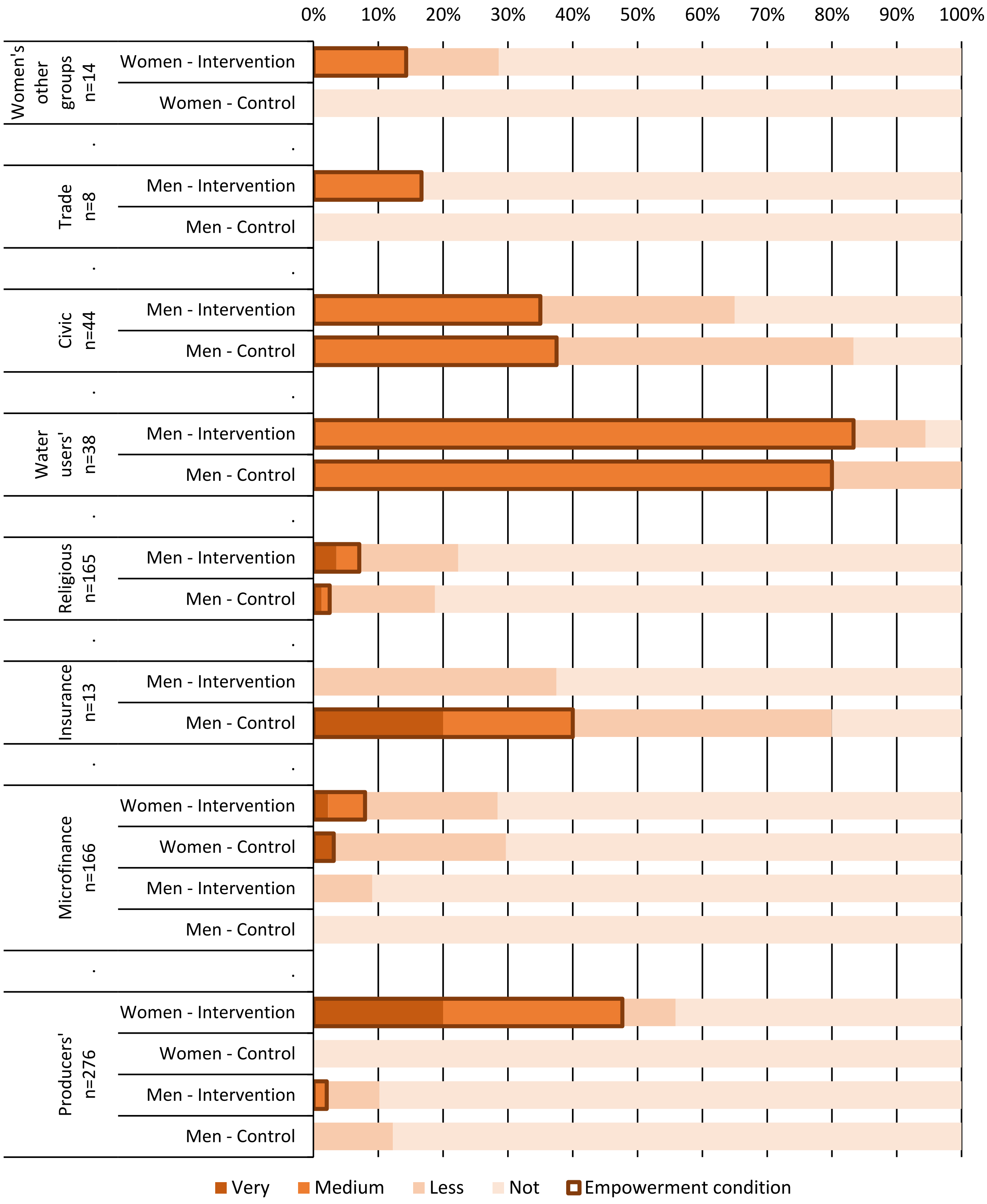


***Supplemental Figure S7 : Perceived influence of community groups to which the respondent is a member, by group type, sex of respondent, and FAARM intervention group***

Due to the small numbers of members of many groups, particularly for women, the table has been simplified. Please note that there was only one man in the forest users' group and he rated the group's influence as medium. The FAARM trial was undertaken in Habiganj District, Sylhet Division, Bangladesh.

***Supplemental Table S10: Impact of the FAARM intervention on Pro-WEAI indicators, by sex of respondent (without covariates)***

| Categorical measures | | | | |
|---|---|---|---|---|
| | Women | | Men | |
| | OR | p-value | OR | p-value |
| *Intrinsic agency* | | | | |
| Intimate partner violence not acceptable | 3.5 | <0.001 | 0.9 | 0.738 |
| Autonomy in income | 1.6 | 0.085 | 2.6 | 0.045 |
| Self-efficacy | 3.1 | <0.001 | 2.2 | 0.002 |
| Respect among household members | 1.0 | 0.994 | 1.4 | 0.380 |
| *Instrumental agency* | | | | |
| Access to and decisions on financial services | 0.6 | 0.348 | 0.4 | 0.388 |
| Ownership of land and other assets | 2.4 | 0.002 | 1.0 | 0.984 |
| Input in productive decisions | 1.1 | 0.752 | 0.9 | 0.804 |
| Control over use of income | 1.6 | 0.123 | 0.6 | 0.066 |
| Visiting important locations | 1.2 | 0.407 | 1.7 | 0.045 |
| Work balance | 0.6 | 0.117 | 0.9 | 0.707 |
| *Collective agency* | | | | |
| Group membership | 12.9 | <0.001 | 1.4 | 0.183 |
| Membership in influential groups | 145.7 | <0.001 | 1.3 | 0.628 |
| Empowered in agency | 6.8 | <0.001 | 1.5 | 0.133 |
| Women's equity with spouse* | | | 3.3 | <0.001 |

| Continuous measures | | | | |
|---|---|---|---|---|
| | Women | | Men | |
| | Coef | p-value | Coef | p-value |
| 3DE score | 1.5 | <0.001 | 0.3 | 0.083 |
| Intrinsic agency | 0.5 | <0.001 | 0.2 | 0.007 |
| Instrumental agency | 0.2 | 0.103 | 0.0 | 0.710 |
| Collective agency | 0.9 | <0.001 | 0.1 | 0.245 |

Note: Results are based on multilevel logistic and linear regression models with random effects on the data collector and cluster levels. Model only includes the sex of the respondent and the treatment effects by sex (given above). The FAARM trial was undertaken in Habiganj District, Sylhet Division, Bangladesh. n=885 except for "Women's equity with spouse" where *n=420

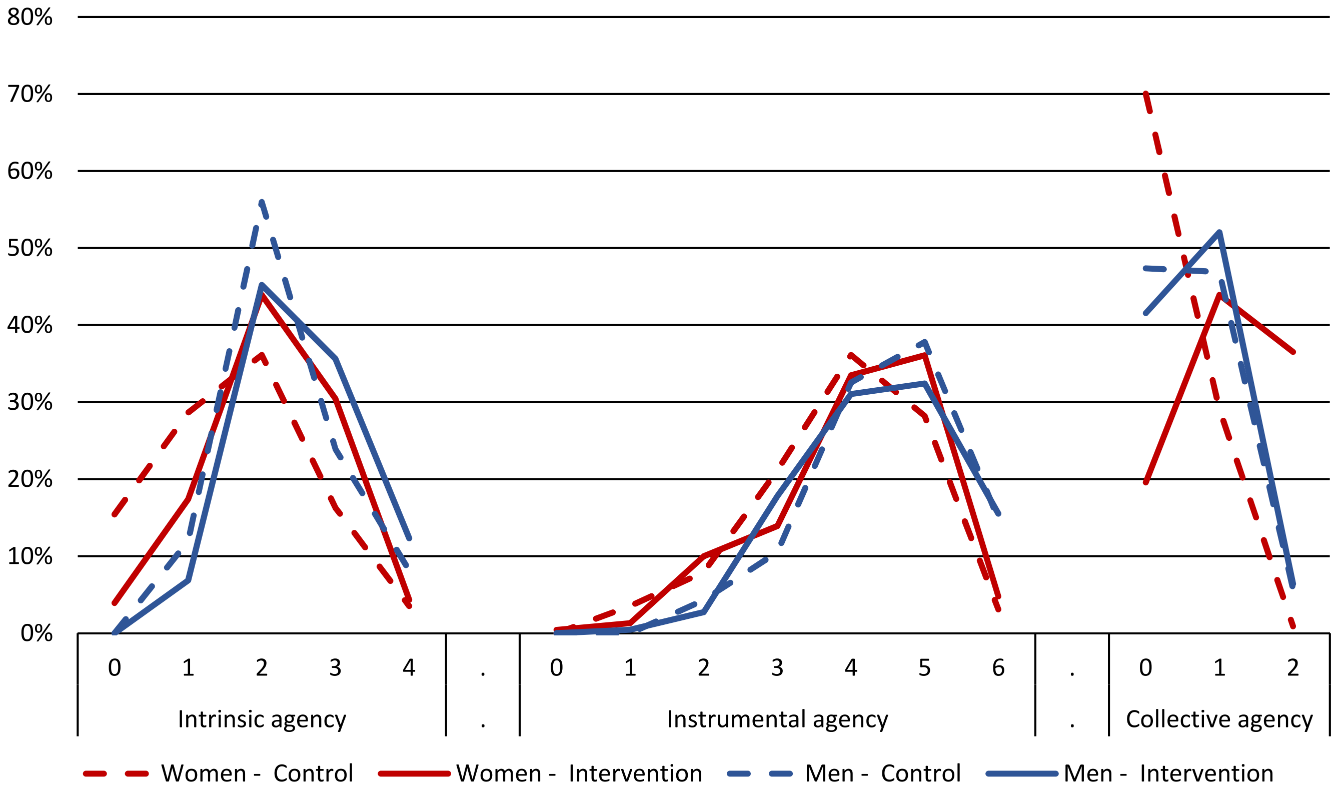


***Supplemental Figure S8: Density plot of the number of indicators in each domain for which empowerment was attained, by sex of respondent and FAARM intervention group***

The proportion of respondents, by sex and intervention, who achieved empowerment on the number of indicators given on the x-axis, by agency domain. Blue is for men and red for women, while the dashed lines are for control group and the solid lines for the intervention group. The FAARM trial was undertaken Habiganj District, Sylhet Division, Bangladesh. n=885

***Supplemental Table S11: Mean number of indicators for which empowerment was attained, by pro-WEAI domain, sex of respondent, and FAARM intervention group***

| | | Women | | | | Men | | | |
|---|---|---|---|---|---|---|---|---|---|
| | Posible range | Control | | Intervention | | Control | | Intervention | |
| | | mean | *(95% CI)* | mean | *(95% CI)* | mean | *(95% CI)* | mean | *(95% CI)* |
| All 3DE indicators | 0-12 | 5.8 | *(5.5-6.1)* | 7.4 | *(7.1-7.7)* | 7.3 | *(7.1-7.6)* | 7.6 | *(7.3-7.8)* |
| Intrinsic agency | 0-4 | 1.6 | *(1.4-1.9)* | 2.1 | *(2.0-2.3)* | 2.3 | *(2.1-2.4)* | 2.5 | *(2.4-2.7)* |
| Instrumental agency | 0-6 | 3.9 | *(3.7-4.1)* | 4.1 | *(3.9-4.3)* | 4.5 | *(4.3-4.7)* | 4.4 | *(4.2-4.6)* |
| Collective agency | 0-2 | 0.3 | *(0.2-0.4)* | 1.2 | *(1.0-1.3)* | 0.6 | *(0.5-0.7)* | 0.6 | *(0.5-0.7)* |

The FAARM trial was undertaken in Habiganj District, Sylhet Division, Bangladesh. n=885

***Supplemental Table S12: Full regression results for the impact of the FAARM intervention on pro-WEAI indicators***

| | Intimate partner violence not acceptable | | Autonomy in income | | Self-efficacy | |
|---|---|---|---|---|---|---|
| | OR | p-value | OR | p-value | OR | p-value |
| Intervention group by sex | | | | | | |
| Intervention (vs control) in men | 0.94 | 0.864 | 2.61 | 0.047 | 2.26 | 0.002 |
| Intervention (vs control) in women | 3.52 | <0.001 | 1.73 | 0.045 | 3.20 | <0.001 |
| Women (vs men) | 0.34 | 0.424 | 0.07 | 0.001 | 0.77 | 0.757 |
| Wealth tercile | | | | | | |
| Low wealth (vs middle) | 1.11 | 0.680 | 1.09 | 0.740 | 0.99 | 0.954 |
| High wealth (vs middle) | 0.79 | 0.449 | 1.24 | 0.481 | 1.08 | 0.743 |
| Hindu household (vs Muslim) | 0.99 | 0.960 | 0.63 | 0.095 | 0.68 | 0.088 |
| Education | | | | | | |
| Partial primary (vs no education) | 1.70 | 0.085 | 1.14 | 0.665 | 1.65 | 0.047 |
| Completed primary (vs no education) | 1.43 | 0.268 | 2.72 | 0.003 | 1.92 | 0.013 |
| Any secondary (vs no education) | 2.40 | 0.005 | 2.12 | 0.017 | 2.17 | 0.002 |
| Respondent age in years | 1.06 | 0.060 | 0.98 | 0.587 | 1.00 | 0.995 |
| Years since marriage | 0.95 | 0.191 | 1.00 | 0.981 | 1.02 | 0.487 |
| Number of household members | 0.91 | 0.224 | 0.94 | 0.430 | 0.95 | 0.419 |
| Nuclear family structure (vs. joint) | 0.73 | 0.293 | 1.13 | 0.662 | 0.78 | 0.297 |
| Number of observations | 885 | | 885 | | 885 | |

| | Respect among household members | | Access to and decisions on financial services | | Ownership of land and other assets | |
|---|---|---|---|---|---|---|
| | OR | p-value | OR | p-value | OR | p-value |
| Intervention group by sex | | | | | | |
| Intervention (vs control) in men | 1.33 | 0.462 | 0.48 | 0.551 | 0.95 | 0.960 |
| Intervention (vs control) in women | 1.03 | 0.937 | 0.72 | 0.623 | 2.64 | 0.001 |
| Women (vs men) | 0.64 | 0.681 | 0.24 | 0.265 | 0.02 | <0.001 |
| Wealth tercile | | | | | | |
| Low wealth (vs middle) | 1.37 | 0.313 | 4.31 | 0.069 | 0.95 | 0.876 |
| High wealth (vs middle) | 0.89 | 0.723 | 0.46 | 0.274 | 1.90 | 0.135 |
| Hindu household (vs Muslim) | 0.59 | 0.126 | 1.06 | 0.944 | 0.42 | 0.010 |
| Education | | | | | | |
| Partial primary (vs no education) | 0.88 | 0.754 | 1.63 | 0.485 | 2.26 | 0.040 |
| Completed primary (vs no education) | 1.81 | 0.113 | 13.67 | 0.029 | 2.20 | 0.055 |
| Any secondary (vs no education) | 2.79 | 0.005 | 9.08 | 0.011 | 4.02 | <0.001 |
| Respondent age in years | 1.00 | 0.915 | 1.10 | 0.328 | 1.02 | 0.644 |
| Years since marriage | 1.00 | 0.946 | 1.07 | 0.595 | 1.15 | 0.024 |
| Number of household members | 1.00 | 0.998 | 0.75 | 0.064 | 0.83 | 0.036 |
| Nuclear family structure (vs. joint) | 0.61 | 0.147 | 1.35 | 0.714 | 0.69 | 0.324 |
| Number of observations | 885 | | 885 | | 885 | |

***Supplemental Table S12: Full regression results for the impact of the FAARM intervention on pro-WEAI indicators (continued)***

| | Input in productive decisions | | Control over use of income | | Visiting important locations | |
|---|---|---|---|---|---|---|
| | OR | p-value | OR | p-value | OR | p-value |
| Intervention group by sex | | | | | | |
| Intervention (vs control) in men | 0.95 | 0.881 | 0.67 | 0.118 | 1.71 | 0.052 |
| Intervention (vs control) in women | 1.18 | 0.549 | 1.81 | 0.042 | 1.12 | 0.615 |
| Women (vs men) | 0.62 | 0.689 | 2.30 | 0.479 | 0.34 | 0.195 |
| Wealth tercile | | | | | | |
| Low wealth (vs middle) | 1.15 | 0.542 | 1.26 | 0.301 | 0.78 | 0.206 |
| High wealth (vs middle) | 1.20 | 0.513 | 0.88 | 0.644 | 0.92 | 0.719 |
| Hindu household (vs Muslim) | 1.24 | 0.400 | 1.41 | 0.136 | 1.11 | 0.636 |
| Education | | | | | | |
| Partial primary (vs no education) | 1.25 | 0.448 | 0.87 | 0.617 | 1.48 | 0.104 |
| Completed primary (vs no education) | 1.35 | 0.325 | 0.83 | 0.524 | 1.11 | 0.682 |
| Any secondary (vs no education) | 1.48 | 0.169 | 1.11 | 0.699 | 1.76 | 0.018 |
| Respondent age in years | 1.04 | 0.102 | 1.03 | 0.115 | 0.96 | 0.059 |
| Years since marriage | 1.07 | 0.061 | 1.03 | 0.402 | 1.07 | 0.012 |
| Number of household members | 0.91 | 0.162 | 0.76 | <0.001 | 1.12 | 0.049 |
| Nuclear family structure (vs. joint) | 1.23 | 0.433 | 0.68 | 0.125 | 1.57 | 0.047 |
| Number of observations | 885 | | 885 | | 885 | |

| | Work balance | | Group membership | | Membership in influential groups | |
|---|---|---|---|---|---|---|
| | OR | p-value | OR | p-value | OR | p-value |
| Intervention group by sex | | | | | | |
| Intervention (vs control) in men | 0.95 | 0.834 | 1.48 | 0.117 | 1.39 | 0.557 |
| Intervention (vs control) in women | 0.66 | 0.153 | 13.97 | <0.001 | 166.82 | <0.001 |
| Women (vs men) | 0.31 | <0.001 | 0.29 | 0.032 | 0.07 | 0.094 |
| Wealth tercile | | | | | | |
| Low wealth (vs middle) | 1.37 | 0.130 | 0.75 | 0.149 | 1.48 | 0.294 |
| High wealth (vs middle) | 1.44 | 0.139 | 0.50 | 0.004 | 0.63 | 0.331 |
| Hindu household (vs Muslim) | 0.72 | 0.128 | 1.47 | 0.079 | 0.83 | 0.671 |
| Education | | | | | | |
| Partial primary (vs no education) | 1.09 | 0.721 | 2.00 | 0.004 | 1.60 | 0.327 |
| Completed primary (vs no education) | 1.08 | 0.779 | 1.32 | 0.266 | 2.04 | 0.152 |
| Any secondary (vs no education) | 1.36 | 0.204 | 2.53 | <0.001 | 3.28 | 0.009 |
| Respondent age in years | 1.02 | 0.251 | 1.00 | 0.818 | 1.03 | 0.354 |
| Years since marriage | 1.08 | 0.006 | 1.02 | 0.492 | 0.97 | 0.536 |
| Number of household members | 0.84 | 0.005 | 0.94 | 0.270 | 0.82 | 0.065 |
| Nuclear family structure (vs. joint) | 0.67 | 0.087 | 0.96 | 0.849 | 0.46 | 0.066 |
| Number of observations | 885 | | 885 | | 885 | |

***Supplemental Table S12: Full regression results for the impact of the FAARM intervention on pro-WEAI indicators (continued)***

| | Empowered in agency | | Women's equity with spouse* | | 3DE score | |
|---|---|---|---|---|---|---|
| | OR | p-value | OR | p-value | Coef. | p-value |
| Intervention group | | | 3.54 | <0.001 | | |
| Intervention (vs control) in men | 1.45 | 0.160 | | | 0.30 | 0.036 |
| Intervention (vs control) in women | 7.67 | <0.001 | | | 1.51 | <0.001 |
| Women (vs men) | 0.17 | 0.004 | | | -1.49 | <0.001 |
| Wealth tercile | | | | | | |
| Low wealth (vs middle) | 1.21 | 0.433 | 0.76 | 0.346 | 0.07 | 0.531 |
| High wealth (vs middle) | 0.82 | 0.468 | 0.52 | 0.063 | -0.11 | 0.442 |
| Hindu household (vs Muslim) | 0.69 | 0.135 | 0.55 | 0.057 | -0.17 | 0.153 |
| Education | | | | | | |
| Partial primary (vs no education) | 1.94 | 0.034 | 0.52 | 0.065 | 0.44 | 0.002 |
| Completed primary (vs no education) | 2.37 | 0.007 | 0.90 | 0.774 | 0.51 | <0.001 |
| Any secondary (vs no education) | 4.13 | <0.001 | 0.48 | 0.034 | 0.97 | <0.001 |
| Respondent age in years | 1.03 | 0.123 | | | 0.02 | 0.137 |
| Years since marriage | 1.03 | 0.379 | 0.99 | 0.695 | 0.04 | 0.008 |
| Number of household members | 0.91 | 0.186 | 0.90 | 0.233 | -0.13 | <0.001 |
| Nuclear family structure (vs. joint) | 0.64 | 0.101 | 1.18 | 0.616 | -0.20 | 0.127 |
| Years husband older than wife | | | 1.00 | 0.881 | | |
| Number of observations | 885 | | 420 | | 885 | |

| | Intrinsic agency | | Instrumental agency | | Collective agency | |
|---|---|---|---|---|---|---|
| | Coef. | p-value | Coef. | p-value | Coef. | p-value |
| Intervention group by sex | | | | | | |
| Intervention (vs control) in men | 0.22 | 0.005 | -0.01 | 0.949 | 0.09 | 0.172 |
| Intervention (vs control) in women | 0.46 | <0.001 | 0.18 | 0.068 | 0.86 | <0.001 |
| Women (vs men) | -0.67 | 0.032 | -0.56 | 0.049 | -0.28 | 0.039 |
| Wealth tercile | | | | | | |
| Low wealth (vs middle) | 0.04 | 0.534 | 0.06 | 0.439 | -0.03 | 0.494 |
| High wealth (vs middle) | -0.01 | 0.873 | 0.07 | 0.436 | -0.16 | 0.005 |
| Hindu household (vs Muslim) | -0.17 | 0.012 | -0.05 | 0.556 | 0.05 | 0.364 |
| Education | | | | | | |
| Partial primary (vs no education) | 0.14 | 0.068 | 0.14 | 0.153 | 0.17 | 0.003 |
| Completed primary (vs no education) | 0.30 | <0.001 | 0.12 | 0.223 | 0.10 | 0.102 |
| Any secondary (vs no education) | 0.40 | <0.001 | 0.34 | <0.001 | 0.24 | <0.001 |
| Respondent age in years | 0.00 | 0.495 | 0.01 | 0.266 | 0.00 | 0.602 |
| Years since marriage | 0.00 | 0.703 | 0.05 | <0.001 | 0.00 | 0.737 |
| Number of household members | -0.02 | 0.160 | -0.08 | <0.001 | -0.02 | 0.117 |
| Nuclear family structure (vs. joint) | -0.11 | 0.113 | -0.03 | 0.716 | -0.05 | 0.348 |
| Number of observations | 885 | | 885 | | 885 | |

Results are based on multilevel logistic and linear regression models with random effects on the data collector and cluster levels. The FAARM trial was undertaken in Habiganj District, Sylhet Division, Bangladesh. *observations are couples (husbands and wives)
